# A KALA-modified lipid nanoparticle containing CpG-free plasmid DNA as a potential DNA vaccine carrier for antigen presentation and as an immune-stimulative adjuvant

**DOI:** 10.1093/nar/gkv008

**Published:** 2015-01-20

**Authors:** Naoya Miura, Sharif M. Shaheen, Hidetaka Akita, Takashi Nakamura, Hideyoshi Harashima

**Affiliations:** Department of Molecular Design of Pharmaceutics, Faculty of Pharmaceutical Sciences, Hokkaido University, Kita 12, Nishi 6, Kita-ku, Sapporo, Hokkaido 060-0812, Japan

## Abstract

Technologies that delivery antigen-encoded plasmid DNA (pDNA) to antigen presenting cell and their immune-activation are required for the success of DNA vaccines. Here we report on an artificial nanoparticle that can achieve these; a multifunctional envelope-type nanodevice modified with KALA, a peptide that forms α-helical structure at physiological pH (KALA-MEND). KALA modification and the removal of the CpG-motifs from the pDNA synergistically boosted transfection efficacy. In parallel, transfection with the KALA-MEND enhances the production of multiple cytokines and chemokines and co-stimulatory molecules via the Toll-like receptor 9-independent manner. Endosome-fusogenic lipid envelops and a long length of pDNA are essential for this immune stimulation. Furthermore, cytoplasmic dsDNA sensors that are related to the STING/TBK1 pathway and inflammasome are involved in IFN-β and IL-1β production, respectively. Consequently, the robust induction of antigen-specific cytotoxic T-lymphoma activity and the resulting prophylactic and therapeutic anti-tumor effect was observed in mice that had been immunized with bone marrow-derived dendritic cells *ex vivo* transfected with antigen-encoding pDNA. Collectively, the KALA-MEND possesses dual functions; gene transfection system and immune-stimulative adjuvant, those are both necessary for the successful DNA vaccine.

## INTRODUCTION

DNA vaccines are the next-generation vaccines that may partially substitute for attenuated vaccines or protein vaccines. In preference to the purified or recombinantly engineered protein-based vaccines, a plasmid DNA (pDNA) molecule encoding an antigen gene can be readily constructed, and can be rapidly amplified in bacteria for mass production. Moreover, genetic engineering of the antigen such as mutation, deletion and fusion is also easily done. Thus, DNA vaccination has great potential for application in infectious disease (i.e. influenza virus) and cancers, where the antigen frequently changes, and differed from one individual to the others.

In terms of applications of DNA vaccines, the choice of the target cells for gene transfection must be taken into consideration (1,2). Among the various types of cells, targeting antigen-presenting cells, in particular, dendritic cells (DCs), is crucial for inducing cellular immunity, which plays a particularly important role in protection from tumor growth and viral infectious diseases (i.e. human immunodeficiency virus, herpes simplex virus) (3–5).

Many efforts, using both viral and non-viral approaches, have been made to improve transgene efficiency against DCs (6). Since the transfection activities of viral vectors are generally more prominent compared to non-viral vectors (6,7), more clinical trials have been initiated using viral vectors such as adenoviruses and modified vaccinia ankara (8). However, clinical trials using viral vector systems have encountered serious adverse effect, including oncogenicity and excess immune responses (9,10). Another drawback in use of viral approaches is referred to as ‘vector-specific immunity’ (7), which is observed in patients who have already generated antibody against the viruses. In this case, they cannot benefit from a vaccination because of their pre-existing immunity to viruses (11,12). Therefore, the development of non-viral methods would be highly desirable as a compliment to the viral approach, in terms of expanding the choice of therapeutic system for use as a DNA vaccine.

Concerning non-viral vectors, we recently reported on the development of an octaarginine (R8)-modified multifunctional envelop-type nanodevice (R8-MEND). This particle is composed of a pDNA-condensed particle formed with a polycation, and its encapsulating liposomal envelops that are modified with R8, a protein transduction domain that induce cellular uptake (13,14). While the R8-MEND exhibited transgene expression in dividing HeLa cells, the corresponding activity in bone marrow-derived dendritic cells (BMDCs) or dendritic cell-derived cell line (JAWSII) was quite poor. In an attempt to enhance the nuclear delivery of pDNA by inducing membrane fusion between the nuclear membrane and the envelope structure of the MEND, the surface of the lipid was modified with KALA, a peptide that adopts an α-helical structure in a physiological environment (15). To accomplish this, we developed a lipid derivative of KALA (stearylated KALA; STR-KALA) that was designed to allow it to be modified on the surface of the lipid envelop (16). As we expected, modifying the surface with STR-KALA drastically improved the transfection efficiencies in JAWSII. Thus, a KALA-modified MEND with a simple structure (KALA-MEND) is also a potent candidate for use as a carrier of pDNA targeting DCs. However, we unexpectedly found that the drastic improvement of the transfection activity by the KALA-modification is not attributed to an enhancement in nuclear transport efficiency, but to the elevation of the intrinsic transcription activity of the preparation in JAWS II cells. (17).

In addition to the transfection of the antigen-encoding genes, the activation of innate immunity is also required to elicit effective adaptive immunity during a vaccination (5,18). Of note, antigen presentation without an innate immune response (for example, lack of co-stimulatory molecules) induces the inactivation of adaptive immunity to the presented antigen (anergy) (19). Very recently, microarray analysis revealed that transfection with the KALA-modified R8-MEND to the JAWSII cells induced a greater perturbation in mRNA expression in host cells in comparison with the KALA-unmodified particle. In particular, a more detailed analysis indicated the transcriptional factors that are greatly responsible for the immune activation (for example, NF-κB and STATs) are significantly activated (17). Collectively, these data indicated that the KALA-MEND simultaneously activates DCs upon delivering pDNA to these cells.

The objective of the present study was to demonstrate the successful application of KALA-MENDs as a DNA vaccine carrier using primary cultured BMDCs. Our initial effort was focused on the functional characterization of KALA-MENDs, in an attempt to refine the encapsulated pDNA. Accumulating evidence has revealed the therapeutic potential of unmethylated CpG motif in DNA, a ligand for the Toll-like receptor (TLR)9 in endosome as an immune-stimulatory adjuvant for the treatment of cancer and infectious diseases (20–24). However, we assumed that the use of CpG-containing pDNA might be undesirable for gene transfection in DCs when used for a DNA vaccine, since CpG sequences that represent in the promoter and open reading frame (ORF) are potently associated with a loss of transgene expression efficiency or sustained gene expression (25,26). Thus, we investigated the impact of removing CpG-motifs on gene expression in BMDCs. As described below, the immunization of the BMDCs that had been pre-transfected with KALA-MEND exhibited a drastic tumor-preventing effect regardless of the presence or absence of the additional adjuvant, even when pDNA that was completely lacking in unmethylated CpG motifs was used. Thus, we subsequently focused on the CpG motif-independent immune stimulative function of KALA-MEND by monitoring the expression profile of cytokine/chemokine production using the conventional adjuvant as a comparison. The mechanisms responsible for this immune activation were also addressed. Finally, we assessed the antigen-presentation and antigen-specific cytotoxic T-lymphoma (CTL) activity, and tumor-suppressive effect conferred by the KALA-MEND in terms of therapeutic output.

## MATERIALS AND METHODS

### Materials

1, 2-dioleoyl sn-glycero-3-phosphoethanolamine (DOPE) and egg phosphatidylcholine (EPC) were purchased from Avanti Polar Lipids, Inc. (Alabaster, AL, USA). 3,3′-dioctadecyloxacarbo-cyanine perchlorate (DiO) and 1,1′-dioctadecyl-3,3,3′,3′-tetramethylindodicarbocyanine,4-chlorobenzenesulfonate salt (DiD) were purchased from Invitrogen (Carlsbad, CA, USA). Lipofectamine^®^ 2000 reagent was purchased from Invitrogen. Poly (dA:dT), ovalbumin (OVA), phosphatidic acid (PA) cholesterylhemisuccinate (CHEMS) and lipopolysaccharide (LPS) were purchased from Sigma (St. Louis, MO, USA). Stearylated octaarginine (STR-R8) and stearylated KALA (STR-KALA) were custom-synthesized by Kurabo (Osaka, Japan) as described previously (15). Synthetic oligodeoxynucleotides containing CpG motif (5′-TCCATGACGTTCCTGATGCT-3′, phosphorotioated) were purchased from Hokkaido System Science (Hokkaido, Japan). Mouse recombinant granulocyte-macrophage colony-stimulating factor (GM-CSF) was purchased from R&D systems (Minneapolis, MN, USA). Phycoerythrin (PE) -labeled anti-mouse CD80 (Cat. No.: 104707, clone: 16-10A1) and CD86 (Cat. No.: 105007, clone: GL-1) antibody was purchased from Biolegend (San Diego, CA, USA). The isotype controls of each antibody were purchased from eBioscience (San Diego, CA, USA). Mouse immunoglobulin G (IgG) 1κ was purchased from Sigma (St. Louis, MO, USA). All other chemicals were commercially available and reagent grade products.

### Plasmid construction

Conventional pDNA encoding luciferase (GL3) was prepared by inserting a fragment encoding for GL3, obtained by the HindIII/XbaI digestion of the pGL3 basic vector (Promega, Madison, WI, USA) into the Hind III/XbaI-digested site of pcDNA3.1 (Invitrogen, Carlsbad, CA, USA) (27). For the preparation of the CpG-free pDNA encoding luciferase, the multiple cloning site of pCpGfree-mcs (Invivogen, San Diego, CA, USA) was preliminarily replaced with a new one that can be digested by 5′-Bgl II-Pvu II-Nco I-Sca I-Xba I-Nhe I-3′ by ligating the hybridized oligonucleotide fragments to the Bgl II/Nhe I digestion of pCpGfree-mcs (pCpGfree-NEWmcs). Thereafter, an insert fragment encoding the CpG-free luciferase plus one CpG motif at just below the stop codon, Luc(+1), was obtained by the Nco I/Nhe I digestion of pORF-Luc::Sh-CpG (Invivogen), and ligated to the Nco I/Nhe I-digested site of pCpGfree-NEWmcs (pCpGfree-Luc(+1)). Finally, the last remaining CpG motif was removed by inserting a CpG motif-free nucleotide fragment into a Dra III/Nhe I-digested site of pCpGfree-Luc(+1), where we refer to it as pCpGfree-Luc(0).

For the construction of CpG-free pDNA encoding OVA, CpG-free insert encoding OVA (Supplementary Figure S1) was custom-synthesized (Invivogen), with CC just above the start codon and AGCTAGC just below the stop codon to allow these sequences to be cleaved by NcoI (CCATGG) and Nhe I, respectively. The insert encoding CpG-free OVA was obtained by the Nco I/Nhe I digestion and ligated to the Nco I/Nhe I-digested site of pCpGfree-NEWmcs (pCpGfree-OVA(0)).

### Preparation of MEND

Each MEND was prepared by the lipid film hydration method as reported previously (30–32). In a typical run, pDNA or ODN (0.1 mg/ml) was condensed with protamine (0.1 mg/ml) in 10-mM HEPES (pH 7.4), at an nitrogen/phosphate (N/P) ratio of 2.2. For the compaction of pDNA with KALA peptide core (pDNA/KALA), pDNA (0.1 mg/ml) was condensed with KALA peptide (1 mM) in 10-mM HEPES (pH 7.4), at an N/P ratio of 3.5. A lipid film was prepared in a glass test tube by evaporating a chloroform and ethanol solution of the lipids, containing DOPE and PA or CHEMS at a molar ratio of 7:2 (total lipid amount: 110 nmol), a composition characterized as an endosome-fusogenic lipid (28). The prepared lipid film was then hydrated with a condensed pDNA solution (200 μl, corresponding to 8.0-μg pDNA) for 10 min at room temperature (0.55 mM of total lipid concentration). In the case of empty liposomes, 10-mM HEPES was used for lipid film hydration. After hydration, the tube was sonicated for 1 min in a bath-type sonicator (AU-25C; Aiwa Co., Tokyo, Japan) to complete the lipid coating of the condensed DNA. Finally, STR-KALA or STR-R8 (final concentration, 10 mol% of total lipid) was added to the MEND solution under vortexing. The diameter and ξ-potential of the MEND and core particle were determined using an electrophoretic light-scattering spectrophotometer (Zetasizer; Malvern Instruments Ltd., Malvern, WR, UK).

### Preparation of BMDCs of mice

Female C57BL/6 (H-2^b^) mice (6–8 weeks old) were obtained from Japan SLC, Inc. (Shizuoka, Japan). The protocol for use of the mice was approved by the Pharmaceutical Science Animal Committee of Hokkaido University. BMDCs were prepared as described previously (29). Briefly, bone marrow cells were cultured overnight in RPMI1640 medium containing 50-μM 2-mercaptethanol, 10-mM HEPES, 1-mM sodium pyruvate, 100-U/ml penicillin-streptomycin and 10% fetal calf serum (FCS). Non-adherent cells were harvested and cultured in the same medium supplemented with 10-ng/ml GM-CSF. On days 2 and 4, non-adherent cells were removed, and the remaining adherent cells were cultured in fresh medium containing 10-ng/ml GM-CSF. On day 6 or 7, non-adherent cells were used in experiment as immature BMDC.

### Transfection to BMDC with the MENDs for evaluating gene expression

For transfection studies, BMDCs (4.0 × 10^5^ cells) were incubated with the MEND (equivalent to 0.4-μg pDNA) in serum-free medium for 30 min or 3 h. Thereafter, a medium containing 10% FCS was added, and the resulting suspension was incubated for an additional 21 h. GM-CSF was added in the medium at 10 ng/ml over through the transfection studies. The cells were then washed and solubilized with the reporter lysis buffer (Promega, Madison, WI, USA). Luciferase activity in the cell lysate was then measured by means of a luminometer (Luminescencer-PSN; ATTO, Tokyo, Japan). The amount of protein in the cell lysate was determined using a Bicinchoninic acid (BCA) protein assay kit (PIERCE, Rockford, IL, USA).

### Transfection to BMDC with the MENDs for evaluating the production of cytokines and chemokines

For transfection studies, BMDCs (4.0 × 10^5^ cells) were incubated with the MEND (equivalent to 0.4-μg pDNA) or Poly (dA:dT) complexed with Lipofectamine^®^ 2000 reagent (0.4 μg: 1.2 μl) for 3 h in serum-free RPMI-1640 medium. In the inhibition experiment, BMDCs were incubated with medium containing BX795 or Ac-YVAD-CMK, an inhibitor of TBK1 or caspase-1 for 30 min before incubation with MEND. Then, RPMI-1640 medium containing 10% FCS was then added, followed by further 3 h of incubation. Throughout the transfection studies, 10-ng/ml GM-CSF was added in RPMI-1640 medium. Thereafter, the concentration of IFN-β, IL-1β, IL-6, IL-27p28, IFN-γ-inducible protein-10 (IP-10), Monokine induced by gamma interferon (MIG), Macrophage inflammatory protein-1β (MIP-1β) and TNF-α in the culture supernatant was measured by enzyme-linked immunosorbent assay (ELISA) supplied by R&D systems (for IL-1β, IL-6, IL-27p28, IP-10, MIG, MIP-1β and TNF-α; Minneapolis, MN, USA) and PBL InterferonSource (for IFN-β; Piscataway, NJ, USA).

### Fluorescence-activated cell sorting analysis

To evaluate the the expression of CD80 and CD86 on the BMDCs, the cells (8.0 × 10^5^ cells) were incubated with the MENDs (equivalent to 0.4-μg pDNA) or LPS (final concentration, 1 μg/ml) for 3 h in serum-free RPMI-1640 medium. RPMI-1640 medium containing 10% FCS was then added, followed by a further incubation for 18 h. Throughout experiments, 10-ng/ml GM-CSF was added in RPMI-1640 medium. Then, the cells (5.0 × 10^5^ cells) were incubated with 5 μg/ml of mouse IgG1κ at 4°C for 30 min. After washing three times with fluorescence-activated cell sorting (FACS) buffer (phosphate buffered saline (PBS) containing 0.5% bovine serum albumin and 0.1% NaN_3_), 5-μg/ml antibodies of PE-labeled anti-mouse CD80, CD86 and each isotype control were added to the BMDCs. The BMDCs were then incubated at 4°C for 30 min. After washing three times with FACS buffer, the BMDCs were analyzed by FACSCalibur™ (Becton Dickinson, Franklin Lakes, NJ, USA).

### Labeling of pDNA

For the visualization of pDNA, the molecule was first labeled using a Mirus Label IT^®^ biotin nucleic acid labeling kit (Mirus Corp., Madison, WI, USA). The pDNA was labeled in the optimized buffer supplied with the kit, but the Label IT solution was mixed at a 1/10 concentration of the recommended protocol by diluting the solution with distilled water. pDNA was incubated for 60 min, and the labeled pDNA was purified by ethanol precipitation. The concentration of biotin-labeled pDNA was adjusted to 0.2 μg/μl. Secondary, fluorescent labeling was done by Qdot^®^ 705 ITK^TM^ Streptavidin Conjugate Kit (Invitrogen). A 50-μl aliquot of the biotin-labeled pDNA solution was then incubated with a 10-μl aliquot of the Qdot^®^ 705 (QD705) streptavidin (final concentration, 0.02 μM) in HEPES (pH 7.4) buffer at room temperature for at least 30 min. After quantum dot (QD) labeling, the excess QD was separated from the labeled DNA by passing the solution through a Sephadex G50 mini column supplied with Mirus Label IT^®^ Kit, which had been pre-equilibrated with HEPES (pH 7.4) buffer.

### Microscopy observation

BMDCs (4.0 × 10^5^ cells) were incubated with MEND, which encapsulated QD705-labeled pDNA prepared as described above, for 2 h in serum-free RPMI-1640 medium. RPMI-1640 medium containing 10% FCS was then added, followed by a further 4 h-incubation. Throughout experiments, 10-ng/ml GM-CSF was added in RPMI-1640 medium. After incubation, the cells were washed twice with RPMI-1640 medium and 20-units/ml Heparin solution, and analyzed using spinning disk confocal microscopy without fixation. Nuclei of cells were stained with Hoechst 33342 for 10 min and acidic compartments were stained with Lysotracker^®^ Green DND-26 (Invitrogen) for 5 min before observation.

Images were acquired by a Nikon ECLIPSE TE-2000-U (Nikon, Tokyo, Japan) equipped with a spinning disk confocal unit, CSU-X1 (Yokogawa Electric Corporation, Tokyo, Japan) and a Nikon Plan Apo 100×/1.40 oil immersion objective (Nikon). Control of the microscopy and acquisition of digital images were performed with the NIS-Elements software (Nikon). Images were captured with an electron multiplier charge device camera (ImagEM; Hamamatsu Photonics, Hamamatsu, Japan). Image analysis was performed by Image Pro Plus (Media Cybernetics, Inc., Rockville, MD, USA). Endosomal escape rate was calculated by the following formula; ((1 − (yellow integrated pixel intensity/red integrated pixel intensity)) × 100).

### Antigen presentation

BMDC cells were transfected using MENDs containing pDNA encoding the OVA plasmid as described above. After 24 h, the transfected DCs were harvested by pipetting, followed by centrifugation at 1600 rpm for 5 min. The transfected BMDCs (2 x 10^5^ cells) were mixed with B3Z T-cell hybridoma (1 × 10^5^ cells) in RPMI 1640 with 10% FCS in 96-well plates, and incubated for 15 h at 37°C (30). The co-cultured cells were then washed with 200-μl PBS and then incubated with 100 μl of chlorophenol red β-D-galactopyranoside buffer (5-mM chlorophenol red β-D-galactopyranoside, 0. 5% NP-40 and 9-mM MgCl_2_ in PBS) for 4 h at 37°C. After the incubation, the absorbance at 595 nm of each well was measured using a micro plate reader (Benchmark Plus; Bio-Rad).

### Anti-tumor effect

In the case of tumor-prevention experiments, pCpGfree-OVA(0) or non-coding pCpGfree-mcs was transfected to the BMDCs by using KALA-MEND. After the transfection for 24 h, BMDCs were harvested and were then administered (4 × 10^5^cells/40 μl) through the footpad on 14 day and 7 day before the tumor inoculation. In a group, the BMDCs were additionally treated with 1 μg/ml of CpG oligonucleotide for 60 min prior to the food-pad injection. Thereafter, 8 × 10^5^ E.G7-OVA cells were subcutaneously injected.

For the evaluation of the therapeutic effect against tumor, individual mice were inoculated with 8 × 10^5^ E.G7-OVA cells. In parallel, pCpGfree-OVA(0) or pCpGfree-Luc(0) was transfected to the BMDCs via the KALA-MEND or the R8-MEND for 6 h. At 7 days and 14 days after tumor inoculation, the mice were immunized with the harvested BMDCs by injection into the footpad (5 × 10^5^cells/40-μl PBS). Tumor volume was calculated by the following formula: (major axis × minor axis^2^) × 0.52.

### *In vivo* CTL assay

*In vivo* CTL assay was performed as described previously (31). Briefly, C57BL/6 mice were subcutaneously immunized with each sample. After 7 days, splenocytes were prepared from naïve C57BL/6 mice and incubated for 30 min at 37°C with the OVA_257–264_ peptide in RPMI-1640 medium containing 50-μM 2-mercaptoethanol, 10-mM HEPES, 1-mM sodium pyruvate, 100-units/ml penicillin-streptomycin and 10% FCS. The OVA_257–264_ peptide-presented splenocytes were then labeled by incubation for 10 min at 37°C with 5-μM CFSE in PBS (CFSE_high_ cells). The naïve splenocytes were labeled by incubation for 10 min at 37°C with 0.5-μM CFSE in PBS (CFSE_low_ cells). CFSE-labeled cells were washed with PBS. A mixture of 5 × 10^6^ CFSE_high_ and 5 × 10^6^ CFSE_low_ cells was intravenously injected into the immunized mice. After 20 h, splenocytes from the immunized mice were collected and single-cell suspensions were analyzed for the detection and quantification of CFSE-labeled cells by FACSCalibur^TM^. The numbers of CFSE_low_ cells were essentially the same in all samples including the non-treated group. The values for lysis were the number of CFSE_high_ cells corrected by the number of CFSE_low_ cells. In this study, non-specific lysis was not observed.

## RESULTS

### Effect of CpG motif in pDNA on the gene expression in BMDC

**Figure 1.**
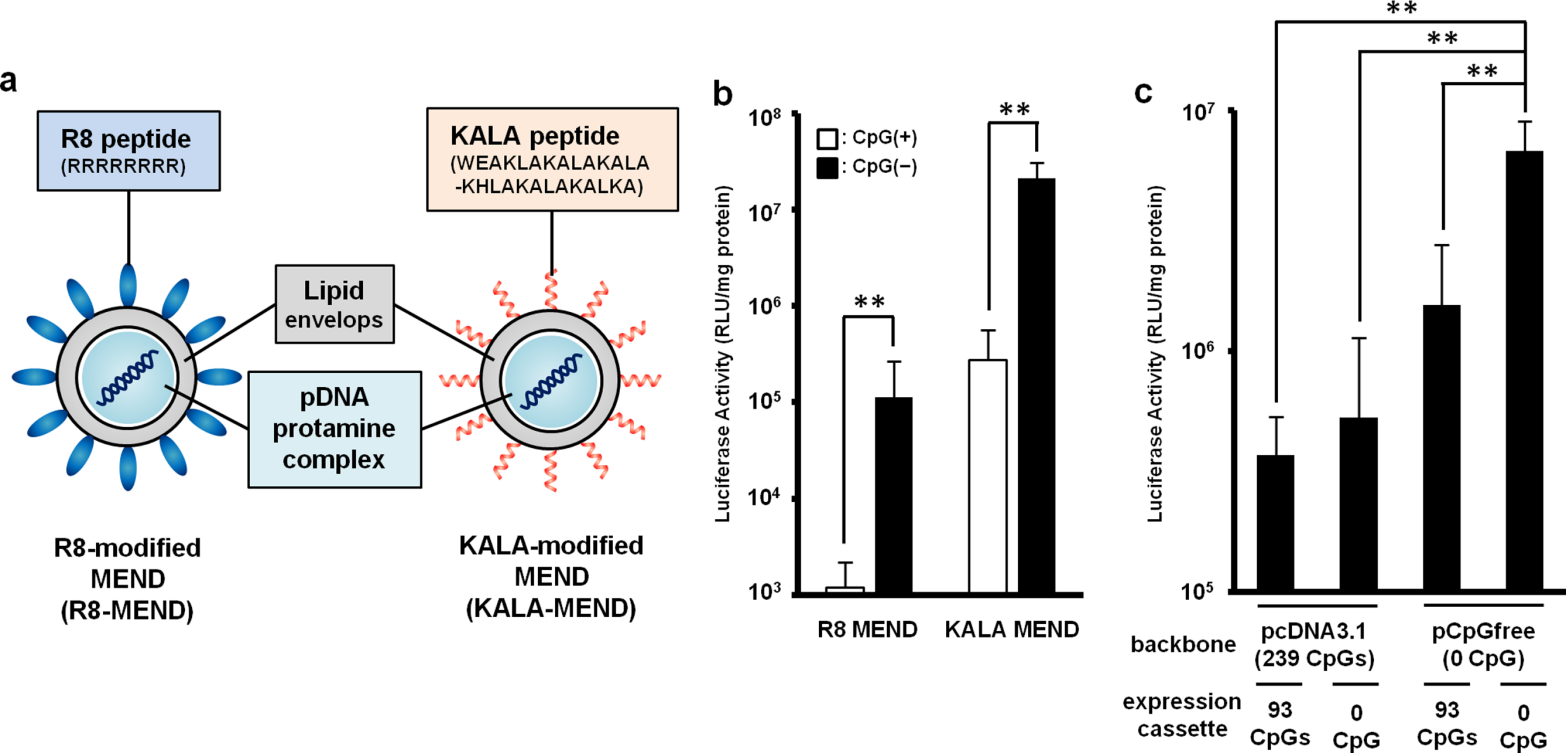
Gene expression efficacies of MENDs in BMDCs. (**a**) Schematic diagram of the R8-MEND (left) and KALA-MEND (right). (**b**) The R8-MEND and KALA-MEND encapsulating a conventional pDNA (pcDNA3.1-Luc; opened bar) or CpG-free pDNA (pCpGfree-Luc(0); closed bar) were transfected to BMDCs. Data were presented as the mean ± SD of three independent experiments. Statistical differences were evaluated by one-way ANOVA, followed by Student's *t*-test (**P < 0.01). (**c**) The transfection activity of KALA-MENDs encapsulating a pDNA with various set of backbone and inserts was also evaluated. Data were presented as the mean ± SD of three independent experiments. Statistical analyses were performed by one-way ANOVA, followed by Bonferroni test. **P < 0.01 versus pCpGfree-Luc(0).

In our previous work (16), we used a mammalian expression vector referred to as pcDNA3.1-Luc (332 CpGs in total pDNA), in which luciferase (GL3) as a reporter gene (93 CpGs in its ORF) is inserted into the pcDNA3.1 (Invitrogen, Carlsbad, CA, USA). In initial experiments, we investigated the impact of the use of CpG-free pDNA (pCpGfree-Luc(0)) on gene transfection activity. In the structure of pCpGfree-Luc(0), the CpG-free luciferase gene (Invivogen, San Diego, CA, USA) was inserted into the pCpGfree-mcs (Invivogen). The pDNA was used in preparing the R8-MEND or KALA-MEND (Figure 1a), which was then transfected to the BMDCs generated from C57BL/6 mice. In an initial study, we prepare these particles using 1.2-dioleoyl sn-glycero-3-phosphoethanolamine (DOPE) and CHEMS as a membrane-fusogenic component of the lipid envelop structure (32,33). The physicochemical characters of the particles were similar regardless of the type of the modified peptide (R8 or KALA) or pDNA used (Table 1). In both types of pDNA, the transfection activity was higher in the KALA-MEND than in the R8-MEND by more than two orders of magnitude (Figure 1b). More importantly, the replacement of the pDNA from pcDNA3.1-Luc to the pCpGfree-Luc(0) resulted in a 50-fold enhancement in gene expression by KALA-MEND. The experiment in Figure 1c was designed to allow us to gain insights into the effect of the CpG deletion in the backbone and/or ORF on the overall enhancement in gene expression. To address this issue, we prepared two additional types of pDNA: a pcDNA3.1 vector encoding the CpG-free luciferase gene (pcDNA3.1-Luc(0); 239 CpGs) and a pCpGfree-mcs vector encoding the CpG-positive luciferase gene (pCpGfree-Luc; 93 CpGs). The removal of the CpG sequences from the backbone and/or the ORF enhanced the gene expression in a synergistic manner (Figure 1c). It is noteworthy that a more prominent enhancement was achieved when CpG was deleted from the backbone region. These collective data show that KALA-MENDs loaded with pCpGfree-Luc(0) represent the best combination for gene delivery in BMDCs.

**Table 1.**
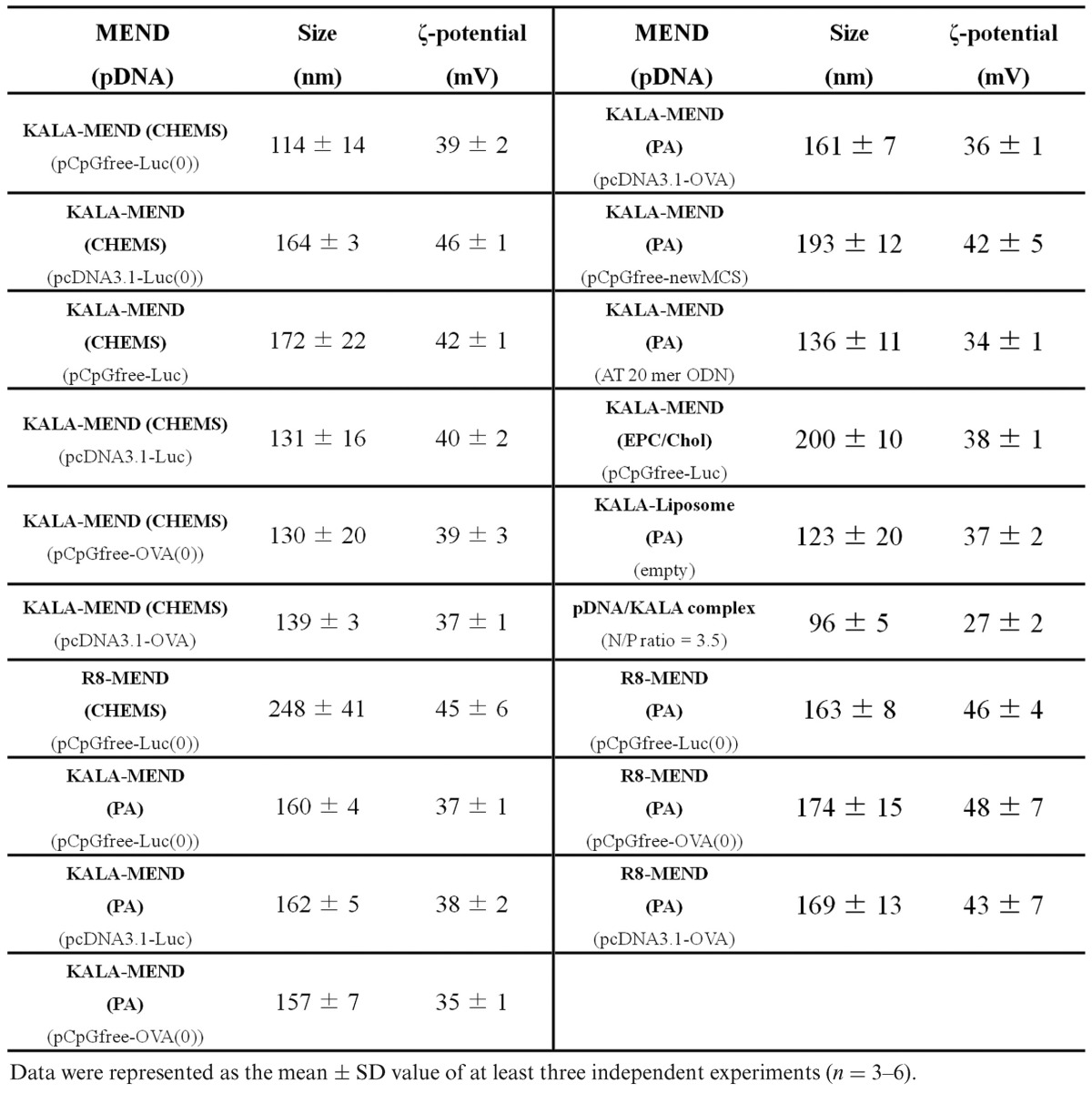
Physicochemical properties of the various MEND

### Tumor-preventing effect by the pre-immunization of BMDCs transfected with the KALA-MEND

**Figure 2.**
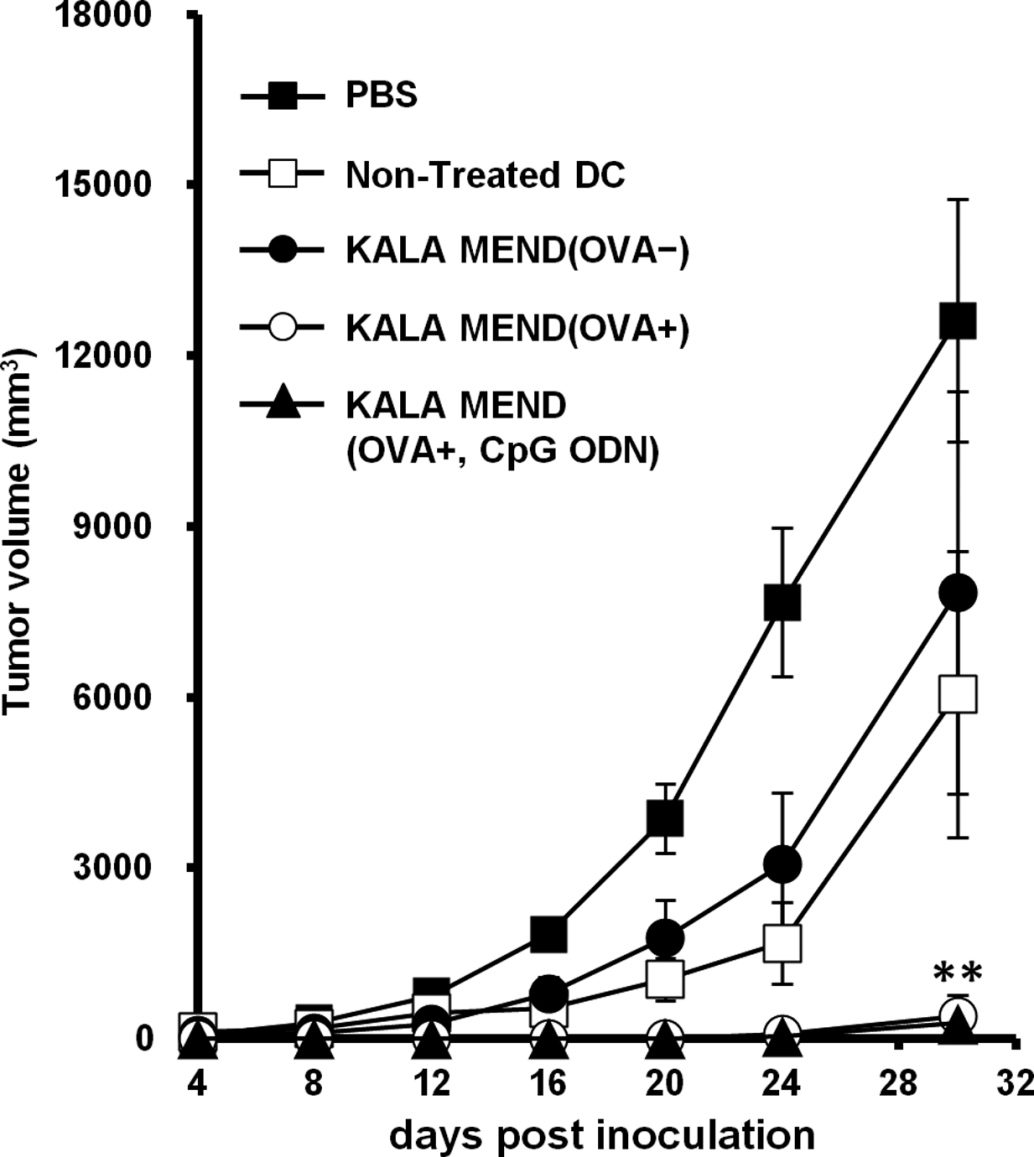
*Ex vivo* prophylactic anti-tumor studies. C57BL/6 mice were immunized twice with BMDCs transfected with pCpGfree-OVA(0) or non-coding pCpGfree-mcs by means of KALA-MEND at 7 days and 14 days before tumor challenging. In one group, BMDCs transfected with pCpGfree-OVA(0) by KALA-MEND were further treated with CpG-oligonucleotide before immunization. As an additional control, PBS, non-treated BMDC and BMDCs treated with CpG-oligonucleotide were injected. The mice were inoculated with E.G7-OVA cells and tumor growth was monitored. The plots represent the mean ± SD (*n* = 5). Statistical differences were evaluated by one-way ANOVA, followed by Dunnett's multiple comparison test (**P < 0.01 versus PBS-treated control).

In order to investigate the pharmacological activity as a DNA vaccine, BMDCs were *ex vivo* transfected with an antigen-encoding gene by KALA-MEND, and its anti-tumor effect was evaluated (Figure 2). As a model, we used E.G7-OVA tumor model, in which OVA was stably expressed as a model antigen. Since the deletion of the CpG motifs is one of the key factors for maximizing gene expression (Figure 1c), we designed the CpG-free ORF sequence encoding a model antigen—OVA. The full sequence is listed in Supplementary Figure S1. The ORF was inserted within a pCpGfree-mcs to produce a CpG-free pDNA encoding OVA (pCpGfree-OVA(0)). Isolated BMDCs were transfected with pCpGfree-OVA(0) by the KALA-MEND for 24 h. After the activation of the cells with or without an adjuvant treatment (CpG-containing oligonucleotide), the immunized cells were administered to mice via the footpad at a dose of 4 × 10^5^ cells/mouse at 7 days and 14 days before tumor challenging. After the inoculation of E.G7-OVA cells, tumor volume was monitored for up to 30 days (Figure 2). When mice were pre-immunized with BMDCs that had been transfected with pDNA free from the antigen-encoding ORF, tumor suppression was comparable to those pre-immunized with the non-treated BMDCs. In contrast, when pCpGfree-OVA(0) was transfected, a drastic decrease in the rate of tumor growth was observed. It is also noteworthy that extensive tumor suppression was achieved regardless of whether an adjuvant (CpG-containing oligonucleotide; CpG-ODN) treatment was used. These data prompted us to assume that the KALA-MEND intrinsically functions to activate BMDCs, similar to an adjuvant.

### Induction of cytokines and co-stimulatory molecules by transfection with the KALA-MEND

**Figure 3.**
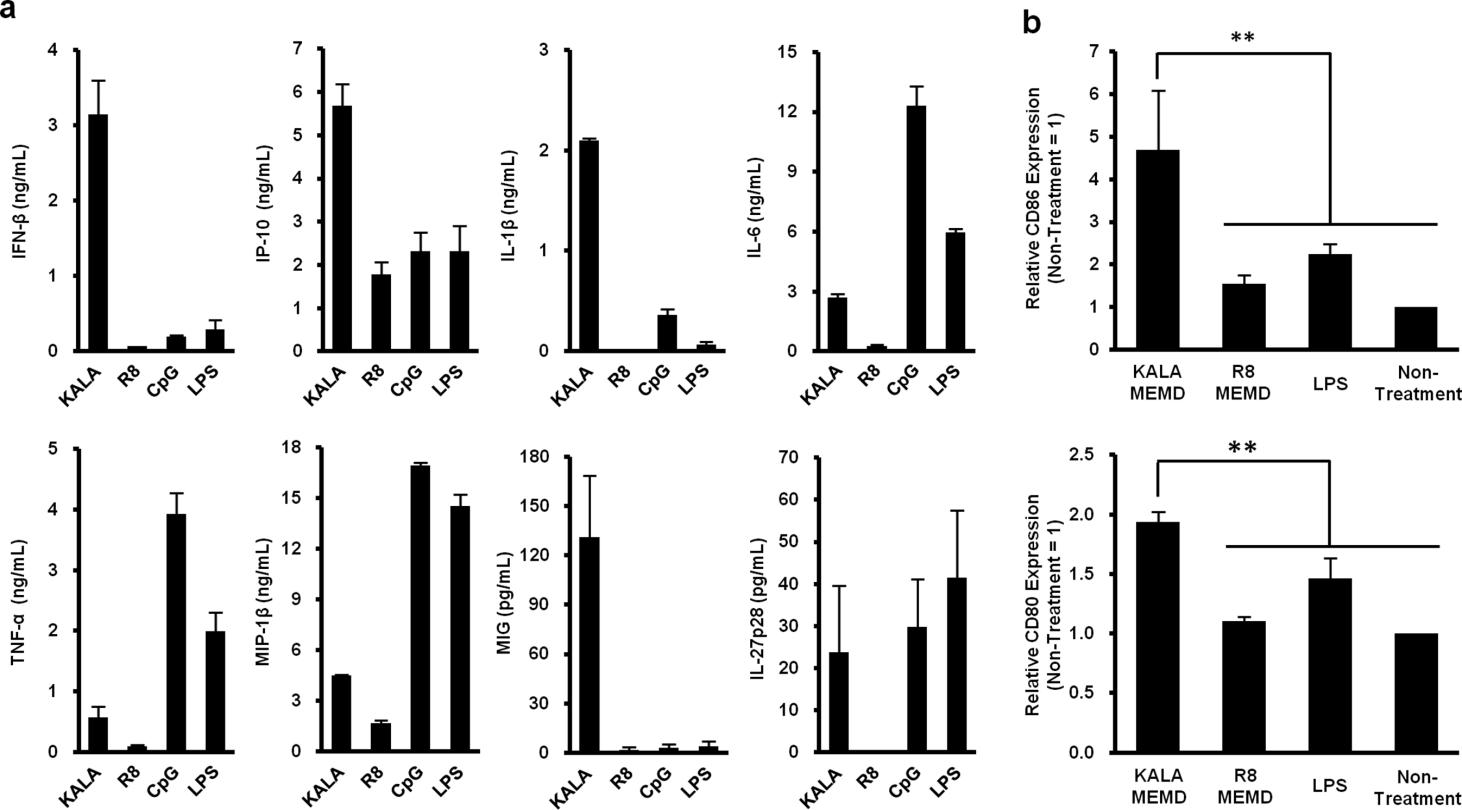
Immuno-stimulatory effect of KALA-MENDs. (**a**) BMDCs (4.0 × 10^5^ cells) were transfected with the KALA- or the R8-MEND (equivalent to 0.4-μg pDNA) or exposed to LPS (100 ng/ml) or CpG ODN (1 μg/ml). After 6 h, the concentrations of several cytokines and chemokines in the supernatant were determined by ELISA. Data are the mean ± SD of three independent experiments. (**b**) BMDCs (8.0 × 10^5^ cells) were transfected with the KALA- or the R8-MEND (equivalent to 0.4-μg pDNA) or exposed to LPS (100 ng/ml). After 21 h, the BMDCs (5.0 × 10^5^ cells) were stained by PE-labeled anti-mouse CD80 and CD86. Data are the means ± SD of three independent experiments. Statistical analyses were performed by the one-way ANOVA, followed by Bonferroni test. **P < 0.01 versus KALA MEND.

We examined the profile of cytokine production from BMDCs after transfection with the KALA-MEND or the R8-MEND encapsulating pCpGfree-Luc(0). In this study, we used DOPE and PA as the composition in the endosome-fusogenic lipid envelope (28) since the KALA-MEND that is composed of DOPE/PA induced higher cytokine production in comparison with those prepared with DOPE/CHEMS (Supplementary Figure S2a). The higher transfection activity and immune-stimulative activities of the KALA-MEND above the R8-MEND were reproducible when MENDs were prepared with DOPA/PA (Supplementary Figure S2b and c). In addition, the activities for the KALA-MEND were drastically higher than for the MEND modified with octa-lysine (K8-MEND). Of note, the amount of protein after the transfection study in the KALA-MEND (78 ± 1% of non-treated DCs) was slightly lower than that in the R8-MEND and the K8-MEND (105 ± 2% and 104 ± 4% of non-treated DCs, respectively). Thus, in some cells, the over-stimulation of the immune-response might damage in DCs transfected by the KALA-MEND. In other words, the lower transfection activities in the R8-MEND or the K8-MEND cannot be explained from the point of view of cytotoxicity. Thus, the BMDCs were transfected with the KALA-MEND or the R8-MEND encapsulating pCpGfree-Luc(0). In a preliminary experiment, the production of the cytokines and chemokines in BMDCs in response to transfection was comprehensively analyzed by means of Multiplex Suspension Array System (Milliplex MAG Kit, Millipore, Billerica, MA, USA, performed by Genetic Lab Co., Ltd., Sapporo, Japan). As shown in Supplementary Figure S3, the production of cytokines and chemokines was more prominent in the case where BMDCs were transfected with the KALA-MEND in comparison with the R8-MEND. Among them, eight cytokines and chemokines (IFN-β, IL-1β, IL-6, IL-27p28, IP-10 (CXCL10), MIG (CXCL9), MIP-1β (CCL4) and TNF-α), whose production was markedly enhanced in the KALA-MEND in preference to the R8-MEND, were selected and their concentration in the medium was measured by an ELISA (Figure 3a). As an additional comparison, the cells were exposed to the conventional adjuvants: LPS (100 ng/ml) or CpG-ODN (1 μg/ml), which are known as ligands for TLR4 and TLR9, respectively. Consistent with the result of a Multiprex Suspention Array, transfection with the KALA-MEND induced a higher degree of cytokine production above the R8-MEND in BMDCs, particularly in IFN-β and IL-1β (∼50- and 230-folds, respectively). Moreover, the level of production of IFN-β, IL-1β, IP-10 and MIG was more prominent in comparison with those stimulated by LPS or CpG ODN, while being not in other cytokines (i.e. IL-6, TNF-α and MIP-1β) (Figure 3a). These data suggest that the profile for cytokine or chemokine production triggered by KALA-MEND was distinct from that for the conventional immune-stimulatory molecules via TLRs (34). We also analyzed the expression level of co-stimulatory molecules (CD80 and CD86) that are known to be induced when the DCs were functionally activated for the T cell stimulation (35). After the transfection of BMDCs with the KALA-MEND or the R8-MEND, or exposure to LPS (1 μg/ml), the BMDCs were stained with an anti-CD80 or anti-CD86 antibody, followed by analysis by flow cytometry (Figure 3b). In parallel with the cytokine expression, CD80 and CD86 molecules on the cell surface were upregulated as the result of the KALA-MEND transfection to a higher level in comparison with LPS treatment, while the effect was marginal in the R8-MEND. These results suggest that the KALA-MEND aids DCs to become adjusted for activating T cells, thus augmenting adaptive immunity in a manner equivalent to an adjuvant.

### Molecular mechanism of immune activation by the KALA-MEND

**Figure 4.**
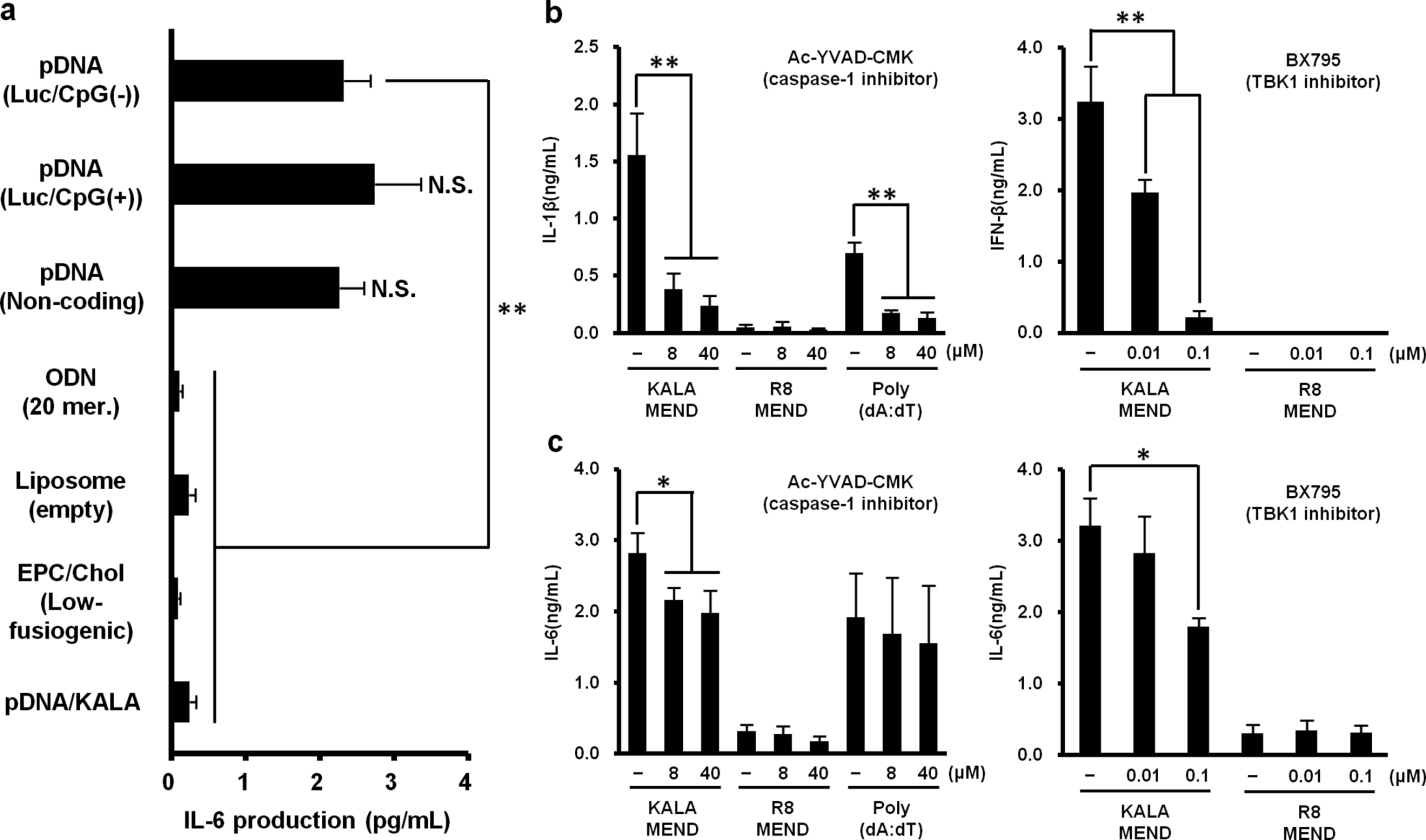
Molecular mechanism of the cytokine expression induced by KALA-MEND. (**a**) BMDCs (4.0 × 10^5^ cells) were transfected with several types of KALA or R8 peptide-modified nanoparticles. (**b,c**) BMDCs (4.0 × 10^5^ cells) were pre-treated with 0.01 or 0.1-μM solutions of BX795 or 8 or 40-μM solutions of Ac-YVAD-CMK for 30 min, then transfected with KALA- or R8-MEND (equivalent to 0.4-μg pDNA) or Poly (dA:dT) conjugated with Lipofectamine^®^ 2000 reagent (0.4 μg:1.2 μl, only Ac-YVAD-CMK treatment). After 6 h, the concentrations of several cytokines in the supernatant were measured by ELISA. Data are mean ± SD of three independent experiments. Statistical analyses were performed by the one-way ANOVA, followed by Bonferroni test. *P < 0.05, **P < 0.01 versus no inhibition group. N.S.: not significant

The mechanism for the immune-stimulation of BMDCs initiated by the transfection with the KALA-MEND was also investigated. First, we attempted to determine which components of the KALA-MEND were responsible for triggering the immune-stimulation. To validate the impact of encapsulated DNA on immune stimulation, pDNA with or without an insert encoding foreign protein (luciferase), or short ODN (AT 20mer.) was encapsulated in the KALA-MEND. Bared liposomes modified with KALA were also prepared. The production of IL-6 was comparable to that achieved in the KALA-MEND, regardless of whether the luciferase-encoding ORF was present or absent, while the corresponding activity was marginal in R8-MEND. Thus, the enhancement in IL-6 production triggered by KALA-MEND is not due to the luciferase protein expressed from the pDNA. Moreover, the most significant finding is that IL-6 production is reduced to the nearly background level when pDNA was replaced to the short ODN or excluded from the particle. Thus, the long length of the DNA appears to be a key trigger for immune-stimulation. IL-6 production was also comparable even when pDNA (3094 bp) encapsulated in the KALA-MEND was digested into three fragments (963, 981 and 1115 bp) (Supplementary Figure S4). Thus, the DNA with a length of ∼1000 base-pairs is still able to function as a trigger for initiating immune-stimulation. Further analyses showed that IL-6 production was impaired when BMDCs were transfected with pDNA that were simply compacted with STR-KALA or the KALA-MEND in which the lipid envelop was prepared with egg phosphatidylcholine (EPC) and Chol (7:3), a non-fusogenic lipid component. These data indicate that the cytoplasmic delivery of a long length of DNA with the aid of the synergic function of endosome-fusogenic lipid envelops and modified KALA peptide is prerequisite for the development of an immune response (Figure 4a).

Recently, an increasing body of evidence has accumulated to indicate that the signaling pathway triggered by cytoplasmic DNA-sensing molecules such as the stimulator of IFN genes (STING)/Tank-binding kinase-1 (TBK1) pathway and absent in melanoma 2 (AIM-2) inflammasome (36–39) play a key role in the dsDNA-triggered production of IFN-β and IL-1β, respectively, in a TLR9-independent manner (40–42). To validate the contribution of these mediators to the immune responses against KALA-MEND, transfection was demonstrated in the presence of the 8 or 40 μM of Ac-YVAD-CMK, an inhibitor of caspase-1 (a key component of inflammasome) or 0.01 or 0.1 μM of BX795, an inhibitor of TBK1/IKKϵ (IκB-kinase epsilon) (43) for 6 h. As a positive control, the Ac-YVAD-CMK inhibited IL-1β production that was triggered by treatment with poly(dA:dT) (Figure 4b). We also recently reported that a BX795 treatment successfully inhibited IFN-β production that was stimulated by the cytoplasmic delivery of cyclic di-GMP, an agonist for the DDX41-mediated STING-TBK1-IRF3 pathway in Raw 267.4 macrophage cells (44). Treatment with Ac-YVAD-CMK resulted in a reduction in the level of IL-1β that was induced by KALA-MEND to <20% in a dose-dependent manner. Moreover, treatment with BX795 also resulted in an IFN-β production being reduced in a concentration-dependent manner (Figure 4b). Collectively, these results correctively indicate that cytosolic DNA sensors that are functionary linked with the STING/TBK1 pathway and inflammasome are responsible for cytokine expression induced by the KALA-MEND. The contribution of the STING/TBK1 pathway and inflammasome to the overall production of IL-6 was also evaluated (Figure 4c). IL-6 production was inhibited by >50% by treatment with BX795, while the inhibition with Ac-YVAD-CMK was significant but marginal. Thus, the STING/TBK1 pathway, rather than the inflammasome pathway, might also contribute to IL-6 production.

### Evaluation of the cellular uptake and intracellular trafficking of KALA-MEND and R8-MEND

To gain further insights into the difference in immune stimulatory activity between the KALA-MEND and the R8-MEND, the cellular uptake and cytoplasmic release of the DNA was investigated. First, the association of the pDNA with the MENDs (non-modified, R8-modified and KALA-modified MEND) was investigated (Supplementary Figure S5). When the particles were directly subjected to 1% agarose electrophoresis, the migration of pDNA was not detectable by steric hindrance. In contrast, once the lipid envelope and pDNA core structure had been disrupted by sodium dodecyl sulphate (1% w/v) and poly-aspartic acid (1 mg/ml) treatment, the pDNA completely migrated, similar to naked pDNA. Thus, the particles were abundantly associated with the particle when they were prepared.

The cellular uptake of the non-modified MEND, the R8-MEND and the KALA-MEND was quantified by FACS (Supplementary Figure S6). The striking observation is that the uptake of the non-modified MEND and the R8-MEND in individual cells was heterogenous, while the R8-modification increased the fraction of cells with high uptake of particles. In contrast, the uptake of the KALA-MEND was more homogenous (with a significantly lower standard deviation in comparison with the non-modified or the R8-MEND; *P* < 0.05). Quantitative analysis revealed that the cellular uptake of the KALA-MEND on average (Geo Mean) was higher than that for the non-modified MEND or the R8-MEND.

**Figure 5.**
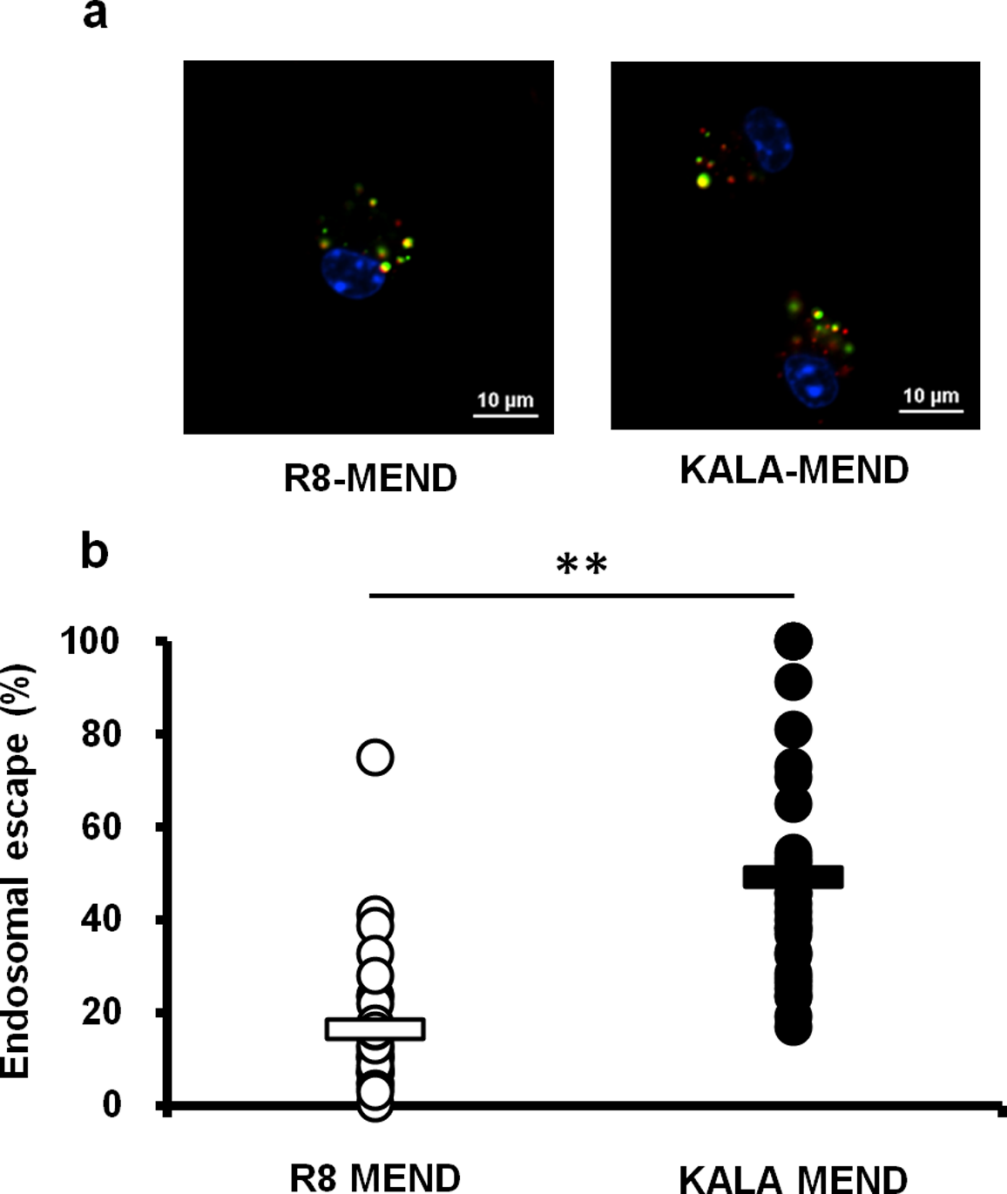
Quantifications of endosomal escape of KALA- or R8-MEND. BMCDs (4.0 × 10^5^ cells) were transfected with the KALA- or the R8-MEND encapsulating QD705-pDNA (equivalent to 0.4-μg pDNA, QD705-pDNA was 50% of whole pDNA; red). (**a**) The BMDCs were observed by spinning-disc confocal microscopy at 6 h after transfection. The nuclei of cells were stained with Hoechst 33342 (blue) and acidic compartments were stained with Lysotracker^®^ Green DND-26 (green) for 10 and 5 min before observation, respectively. Bars = 10 μm. (**b**) Quantification of endosomal escape of KALA- or R8-MEND. Endosomal escape rate was calculated by the following formula: ((1 − (yellow integrated pixel intensity/red integrated pixel intensity)) × 100). Each dot represents the calculated endosomal escape rate of respective cells. Bars show mean (*n* = 30). Statistical analysis was performed by Student's *t*-test. **P < 0.01.

The endosomal escape of pDNA was visualized by intracellular imaging using a fluorescence microscope equipped with a confocal scanning unit (CSU-X1). Since the cytoplasmic recognition of DNA is a plausible trigger for the immune response described above, we assumed that the KALA-MEND was intimately involved in the delivery of pDNA to the cytoplasm and that this process was more efficient than that for the R8-MEND. To validate this hypothesis, we analyzed the co-localization of QD-labeled pDNA in acidic compartments (late endosome/lysosomes) stained with Lysotracker Green at 6 h after transfection. The signals derived from QD705 and Lysotracker Green were pseudo-colored in red and green, respectively. As a result, the QD705-labeled pDNA transfected by the KALA-MEND was less co-localized with acidic compartments than those for the R8-MEND (Figure 5a). Endosomal escape efficiency was quantified in 30 independent cells in terms of the percent of integrated pixel intensity of pDNA free from the co-localization with acidic compartment to the total integrated pixel intensity of pDNA (Figure 5b). As a result, the endosomal escape efficiency of pDNA delivered by the KALA-MEND was significantly higher than that for the R8-MEND.

Finally, the association of the QD705-pDNA with DiO-labeled MENDs in the cells was evaluated (Supplementary Figure S7). While a fraction of the pDNA free from the colocalized with MENDs was detected, the major part of the pDNA was co-localized with the MENDs at the initial phase of the transfection. Thus, efficient endosomal escape was achieved by virtue of the endosome-fusogenic lipid and KALA peptide on the surface.

Thus, a function of KALA as an inducer of cytoplasmic release of pDNA, concomitant with the fusogenic nature of envelop structure in MEND with endosome, is one of the mechanisms that explains the prominent innate immune response.

### MHC class-I antigen presentation and *in vivo* CTL assay

**Figure 6.**
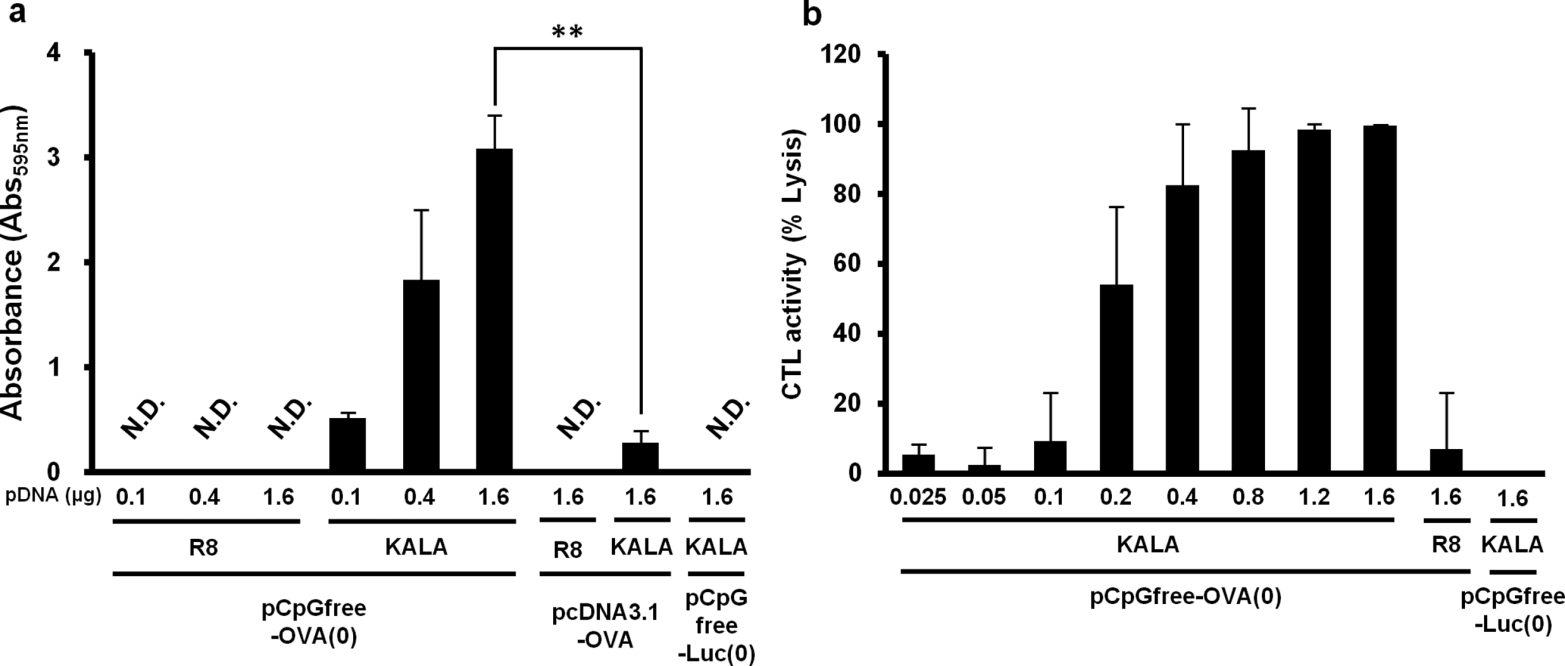
MHC class-I restricted antigen presentation and *in vivo* CTL assay. (**a**) BMDCs were transfected with pcDNA3.1-OVA, pCpGfree-OVA(0) or pCpGfree-Luc(0) (KALA-MEND only) by means of R8-MEND or KALA-MEND. Transfected cells were co-cultured with a B3Z T-cell hybridoma for 15 h at 37°C. The co-cultured cells were then incubated with chlorophenol red β-D-galactopyranoside buffer for 4 h at 37°C. The absorbance at 595 nm was used as an index for antigen-presentation activity. The absorbance observed in non-treated BMDCs was subtracted from each group. Data are mean ± SD of three independent experiments. Non-detected (N.D.)means under the detection limit. Statistical analyses were performed by the one-way ANOVA, followed by Bonferroni test. **P < 0.01 versus pcDNA3.1-OVA. N.S. means non-significance. (**b**) C57BL/6 mice were immunized with BMDCs transfected with KALA-MEND (encapsulating pCpGfree-OVA(0) or pCpGfree-Luc(0)) or R8-MEND (encapsulating pCpGfree-OVA(0)). CTL activities were measured 1 week after immunization. Data are mean ± SD of at least three independent experiments.

To evaluate antigen presentation activity, pCpGfree-OVA(0) was transfected into BMDCs using the R8-MEND or the KALA-MEND. Thereafter, the transfected BMDCs were co-cultured with a B3Z T-Cell hybridoma, a reporter cell that produces the lacZ protein in response to the recognition of the OVA epitope displayed on MHC class-I molecules (30). As a result, significant antigen presentation was observed when pCpGfree-OVA(0) was transfected with the KALA-MEND, while this activity was below the detection limit in the R8-MEND (Figure 6a). Also, the use of pDNA that contains a large number of CpG motif (pcDNA3.1-OVA) or that lacks the OVA-encoding ORF (pCpGfree-Luc(0)) resulted in a diminution in antigen presentation activity to the background level. This result showed that the KALA-MEND encapsulating the antigen-encoding pDNA that is free from CpG motif is a highly potent carrier for the antigen presentation to MHC class-I molecules.

In order to confirm the induction of antigen-specific cellular immunity, we measured the CTL activities after the immunization of BMDC that had been transfected by pCpGfree-OVA(0) with the KALA-MEND or the R8-MEND. As another control, BMDCs were transfected with the KALA-MEND encapsulating the luciferase-encoding pDNA (pCpGfree-Luc(0)) as an antigen-free pDNA. Mice were immunized with the transfected BMDC via footpad administration at a dose of 5.0 × 10^5^ cells. CTL activities were measured at 7 days after the immunization. As a result, BMDC transfected with the KALA-MEND elicited more potent CTL activity than that for the R8-MEND (Figure 6b). Of note, the ED_50_ of pDNA for CTL activity was ∼0.2-μg pDNA, and the activity reached >90% when 0.8 μg of pDNA was used. In contrast, when the pCpGfree-OVA(0) was transfected with the R8-MEND, or when pCpGfree-Luc(0) was, in turn, transfected with the KALA-MEND, the CTL activity was nearly background level even when 1.6-μg pDNA was transfected. These results suggest that the KALA-MEND induced antigen-specific cellular immunity.

### Tumor-treatment effect by the post-immunization of BMDCs transfected with KALA-MEND

**Figure 7.**
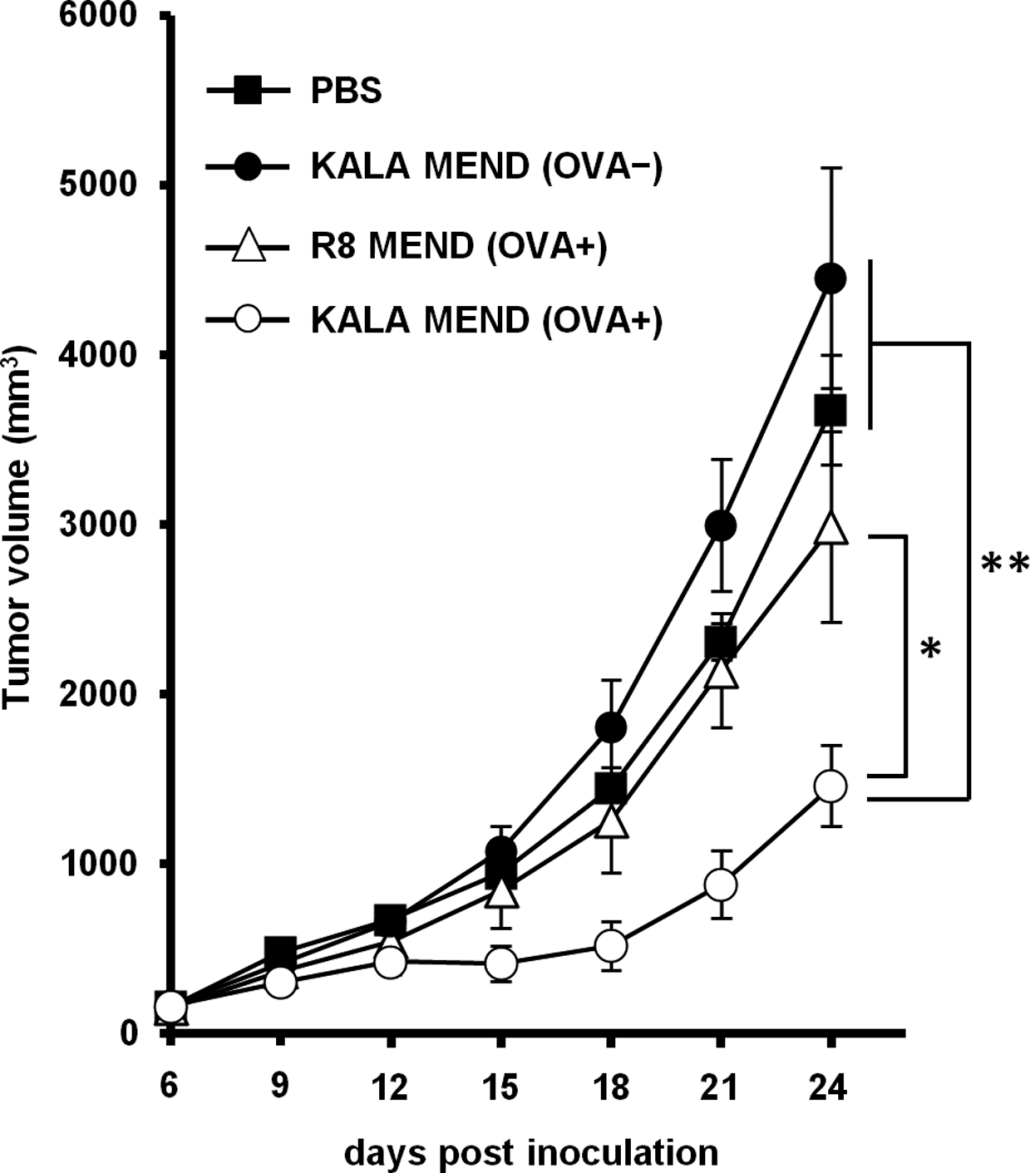
*Ex vivo* therapeutic anti-tumor studies. C57BL/6 mice were immunized twice with BMDCs transfected with pCpGfree-OVA(0) or pCpGfree-Luc(0) by means of the KALA-MEND or the R8-MEND at 7 days and 14 days after tumor challenging. As a control, PBS and non-treated BMDC were injected. Tumor growth was monitored up to 24 days after tumor inoculation. The plots represent the mean ± SEM (*n* = 5). Statistical differences were evaluated by one-way ANOVA, followed by Dunnett's multiple comparison test (**P < 0.01, *P < 0.05 versus KALA-MEND (OVA+)).

Finally, the therapeutic effect in suppression of tumor growth was evaluated. In this study, mice were preliminary inoculated with E.G7-OVA cells. Seven days after tumor challenging (∼150 mm^2^ in tumor size), the BMDCs that had been transfected with KALA-MEND or R8-MEND encapsulating pCpGfree-OVA(0) or pCpGfree-Luc(0) was immunized subcutaneously. At 14 days after the tumor inoculation, the BMDCs that had been transfected with the same types of MENDs were again immunized (Figure 7). As a result, tumor growth was significantly suppressed in the case of BMDCs transfected with pCpGfree-OVA(0) by KALA-MEND, although the R8-MEND encapsulating pCpGfree-OVA(0) or the KALA-MEND encapsulating pCpGfree-Luc(0) poorly inhibits tumor growth. Consequently, the KALA-MEND is applicable for use in cancer therapy even when the tumor is in growing phase.

## DISCUSSION

We report herein on the development of a nanotechnology with the potential for use as a DNA vaccine, which results in an efficient gene expression and simultaneous stimulation to BMDCs to a level sufficient for antigen presentation. As a consequence, the *in vivo* immunization of the BMDCs caused the activation of antigen-specific CTL and a prophylactic and/or therapeutic effect against tumor growth. Key factors in this success are the use of a CpG-free pDNA and a fusogenic peptide: KALA, as a surface-modified peptide.

While some studies reported that the removal of CpG motifs, a ligand for TLR9, resulted in a decrease in gene expression *in vitro* culture cells (CHO and 293) (45) or in electroporation-mediated intra-muscular gene transfer (46), CpG-free pDNA was generally considered to be a promising gene cargo allowing the efficient and particularly persistent gene expression in the lung or liver-targeting gene delivery (26,47–49). Since, these desired effects in the use of CpG-free pDNA were accompanied by a decrease in cytokine production, avoiding immune-stimulation is considered to be a key factor for efficient transgene expression, especially in the case of immune-responsive cells. Based on this background information, we investigated the impact of the use of CpG-free pDNA on transfection efficiency. As expected, the use of pCpGfree-Luc(0) generally improved transgene expression by one to nearly two orders of magnitude in comparison with pcDNA3.1-Luc in BMDCs (Figure 1b). Transgene expression was synergistically enhanced when the CpG-motifs were removed from the backbone of the pDNA and also from the ORF. Therefore, reducing the CpG-motifs from the pDNA is also a key factor in terms of maximizing transgene expression (Figure 1b). Nevertheless, the most mysterious point is that the highest transfection activity of CpG-free pDNA cannot be explained by the avoidance of immune-stimulation, since the multiple inflammatory cytokine was produced at comparable levels, even when CpG-free pDNA was used (Figure 3). One of the proposed mechanisms for the transcriptional suppression of the pDNA (50) as well as genomic DNA involves the methylation of the cytosine nucleotides in the CpG motif (51). Thus, a pDNA constructed with CpG-free human elongation factor 1α (EF1α) promoter used in the pCpGfree-Luc(0) and/or the ORF might be more resistant to gene silencing compared with a set of human cytomegalovirus (CMV) immediate-early enhancer and promoter used in pcDNA3.1-Luc. This hypothesis is consistent with the fact that EF1α promoter is known to be effective for cells in which the CMV promoter functions poorly, such as embryonic stem cells (52).

Using the KALA-MEND, the highest transfection activity was achieved in BMDCs (Figure 1b). While the mode of action of KALA from the point of view of inducing transgene expression remains elusive. The cellular uptake of the KALA-MEND was more homogenous and, on average, higher than that of the non-modified MEND or the R8-MEND (Supplementary Figure S6). However, the uptake of the KALA-MEND was higher than that for the R8-MEND by a factor of nearly 3-fold. Thus, the uptake process cannot explain the >2 orders of magnitude higher transfection activity. Thus, the drastic enhancement in the transfection activity in the KALA-MEND can be attributed to the intracellular process.

As to the functional peptides used in modifying the MENDs, the transfection activity of the K8-MEND was much less than that of the KALA-modified particle, similar to the R8-modified particle. Thus, the high transfection activity and adjuvant effect in the case of the KALA-modified particle cannot be simply explained by the lysine-rich properties of the KALA peptide; the secondary structure of KALA is important for these activities. Meanwhile, we previously revealed that the transfection activity of a particle modified with GALA, another type of amphipathic peptide, was poor in JAWS II cells, a DC-derived cell line (17). As a result, the stimulative activity in transfection activity in GALA was much less than that for KALA. Thus, collectively, these data indicate that the amino acid sequence and the presence of an α-helical structure are both essential for the dual function (gene transfection and adjuvant function) of KALA. In contrast, the microarray analysis and intracellular location of the particle in DC-derived JAWSII cells allowed us to hypothesis that the enhanced gene expression can be attributed to the activation of transcription, that is, in turn closely related to the switch-on of the immune-stimulative pathway (17). In the present study, cytoplasmic delivery of a long length of DNA with the aid of the synergic function of endosome-fusogenic lipid envelops and modified KALA peptide is prerequisite for cytokine production (Figure 3a). In addition, a recent report indicated that STING activation is also elicited by the perturbation of endosome destabilization in viral infection (53). Thus, the fusion of the KALA-MEND with endosome also inherently participates in immune-stimulation and subsequent transgene expression. The KALA-assisted enhancement in the cytoplasmic release of pDNA was also supported by the confocal laser scanning microscopy observations (Figure 5). However, the R8-MEND showed no cytokine production, even though a significant portion of pDNA was released from the endosome. Thus, the all-or-none pattern of cytokine production cannot be explained by the efficiency of endosomal escape.

Meanwhile, for efficient gene expression and subsequent antigen presentation, the nuclear delivery of pDNA is prerequisite after endosomal escape occurs. Since the pDNA was encapsulated in the endosome-fusogenic lipid envelope and then further modified with KALA, a fusogenic peptide, it is plausible that the pDNA might have been released to the cytoplasm as a pDNA/protamine core particle (N/P = 2.2) via membrane fusion with the endosomal membrane. Based on this assumption, the pDNA might enter the nucleus as a particle aided by the nuclear-localization signals in protamine. Alternatively, transcription factors assist the nuclear delivery of pDNA in the naked form after it binds to the specific sequence in pDNA in the cytoplasm as demonstrated previously (54).

As to innate immunity, the induction of cytokines and chemokines is a key event for the activation of immune reactions (55,56). One of the most important findings in the present study is that transfection with the KALA-MEND induces the production of various cytokines and chemokines from BMDCs, the pattern of those was differed from the conventional adjuvants (LPS or unmethylated CpG-ODN) (Figure 3a). The adjuvant activity of the KALA-MEND is also reinforced by the fact that immunization of antigen-transfected BMDCs using the KALA-MEND completely blocked tumor growth for periods of up to 20 days after tumor inoculation without combinatorial treatment with an adjuvant (Figure 2). Among cytokines induced by KALA-MEND, IL-6, IFN-β and IL-1β are key modulators for DCs for the effective induction of adaptive immunity (57,58). In addition, the appropriate differentiation of CD4^+^ T cells is essential for the induction of cellular immunity. IFN-β possesses multiple immune functions. First, it induces Th1 differentiation in CD4^+^ T cell (59). Also, IFN-β elicits BMDCs to become feasible to activate the cross-priming of CD8^+^ T cell (60–62) and induce the expression of maturation markers, such as CD80, CD86, CD40 and MHC molecules (63), and the receptors for chemokines (64). In fact, CD80 and CD86 molecules were effectively upregulated as the result of transfection with the KALA-MEND (Figure 3). Furthermore, MHC class-I-mediated antigen presentation and antigen-specific CTL were also effectively induced by the KALA-MEND transfection (Figure 6). In addition to IFN-β, KALA-MEND induced the production of IL-27, a cytokine that is known to induce Th1 cells (65), and chemokines such as MIG (CXCL9) and IP-10 (CXCL10) which are known to contribute to the Th1 differentiation of naïve CD4^+^ T cell in lymph nodes through their receptor (CXCR3) (66,67). In addition to these chemokines, the KALA-MEND also induced MIP-1β that provides an attractive force for activated T cell (68). Collectively, the KALA-MEND appears to be a highly potent activator of immune responses forward to CD4^+^ T cell-mediated cellular immunity.

Using inhibitors of TBK1/IKKϵ and caspase-1, we revealed that the STING/TBK1 pathway and inflammasome play crucial roles in the induction of IFN-β and IL-1β, respectively, after the treatment of the BMDCs with the KALA-MEND (Figure 4b and c). TBK1 participates in multiple signaling pathways including immune responses, autophagy, proliferation and related phenomena (69). In innate immunity, TBK1 is essential for IRF-3-dependent type-I IFN production mediated by intracellular double-stranded B-DNA (40). However, the issue of specifically how the STING/TBK1 pathway is activated in response to cytoplasmic DNA delivered by the KALA-MEND remains elusive, since STING only senses cyclic dinucleotide, and not double-stranded DNA (70). Recently, additional molecules have been discovered as an additional factor in recognizing dsDNA for STING activation; the DNA-dependent activator of IFN regulatory factors (DAI) (71), gamma-interferon-inducible protein 16 (IFI16) (72), DDX41 (73) and meiotic recombination 11 (MRE11) (74) are included in this group. Moreover, a very recent study showed that cytosolic DNA was recognized by cyclic GMP-AMP (cGAMP) synthase followed by the production of cGAMP, which stimulates STING/TBK1 pathway (75). Further analyses using BMDCs isolated from knock-out mice lacking these proteins will be needed to clarify the responsible sensor for cytoplasmic DNA delivered by the KALA-MEND. Meanwhile, caspase-1, an activator of mature-IL-1β by the cleavage of pro-IL-1β (76), is a component of inflammasome, which recognizes pathogen-associated molecular patterns and danger-associated molecular patterns. Among the inflammasome, the AIM-2 inflammasome is responsible for cytosolic DNA recognition, followed by IL-1β maturation (38). Therefore, it is assumed that the AIM-2 inflammasome is a key mediator of IL-1β production by the KALA-MEND. In conclusion, we succeeded in the development of a carrier for the anti-cancer DNA vaccine, which has dual function in DNA vaccines: an efficient gene carrier for the antigen-encoding pDNA and as an adjuvant triggered by the cytoplasmic delivery of pDNA via a TLR9-independent mechanism. From the standpoint of gene expression and immune-stimulation, the KALA-MEND can be considered to be an artificial virus.

## SUPPLEMENTARY DATA

Supplementary Data are available at NAR Online.

SUPPLEMENTARY DATA
