# Supplementary material for: A KALA-modified lipid nanoparticle containing CpG-free plasmid DNA as a potential DNA vaccine carrier for antigen presentation and as an immune-stimulative adjuvant
Source: Nucleic Acids Res. 2015 Jan 20;43(3):1317–31. doi: 10.1093/nar/gkv008 (PMC4330373; doi:10.1093/nar/gkv008)
Supplement: SUPPLEMENTARY DATA [file supp_43_3_1317__index.html]

A KALA-modified lipid nanoparticle containing CpG-free plasmid DNA as a potential DNA vaccine carrier for antigen presentation and as an immune-stimulative adjuvant — SUPPLEMENTARY DATA 

# A KALA-modified lipid nanoparticle containing CpG-free plasmid DNA as a potential DNA vaccine carrier for antigen presentation and as an immune-stimulative adjuvant

## SUPPLEMENTARY DATA

**Files in this Data Supplement:**

- Supplementary Figures
