# Supplementary material for: A KALA-modified lipid nanoparticle containing CpG-free plasmid DNA as a potential DNA vaccine carrier for antigen presentation and as an immune-stimulative adjuvant
Source: Nucleic Acids Res. 2015 Jan 20;43(3):1317–31. doi: 10.1093/nar/gkv008 (PMC4330373; doi:10.1093/nar/gkv008)
Supplement: SUPPLEMENTARY DATA [file supp_gkv008_nar-02786-f-2014-File010.pdf]

Figure S1

TTAATTAAAATTATCTCTAAGGCATGTGAACTGGCTGTCTTGGTTTTCATCTGTACTTCATCTGCTACCTCTGTGACCTGAAACATA
TTTATAATTCCATTAAGCTGTGCATATGATAGATTTATCATATGTATTTTCCTTAAAGGATTTTTGTAAGAACTAATTGAATTGATAC
CTGTAAAGTCTTTATCACACTACCCAATAAATAATAAATCTCTTTGTTCAGCTCTCTGTTTCTATAAATATGTACCAGTTTTATTGTT
TTTAGTGGTAGTGATTTTATTCTCTTTCTATATATATACACACACATGTGTGCATTCATAAATATATACAATTTTTATGAATAAAAAAT
TATTAGCAATCAATATTGAAAACCACTGATTTTTGTTTATGTGAGCAAACAGCAGATTAAAAGAATTCCTGCAGGAGTCAATGGGA
AAAACCCATTGGAGCCAAGTACACTGACTCAATAGGGACTTTCCATTGGGTTTTGCCCAGTACATAAGGTCAATAGGGGGTGAG
TCAACAGGAAAGTCCCATTGGAGCCAAGTACATTGAGTCAATAGGGACTTTCCAATGGGTTTTGCCCAGTACATAAGGTCAATG
GGAGGTAAGCCAATGGGTTTTTCCCATTACTGACATGTATACTGAGTCATTAGGGACTTTCCAATGGGTTTTGCCCAGTACATAA
GGTCAATAGGGGTGAATCAACAGGAAAGTCCCATTGGAGCCAAGTACACTGAGTCAATAGGGACTTTCCATTGGGTTTTGCCCA
GTACAAAAGGTCAATAGGGGGTGAGTCAATGGGTTTTTCCCATTATTGGCACATACATAAGGTCAATAGGGGTGACTAGTGGAG
AAGAGCATGCTTGAGGGCTGAGTGCCCCTCAGTGGGCAGAGAGCACATGGCCCACAGTCCCTGAGAAGTTGGGGGGAGGGGT
GGGCAATTGAACTGGTGCCTAGAGAAGGTGGGGCTTGGGTAAACTGGGAAAGTGATGTGGTGTACTGGCTCCACCTTTTTCCC
CAGGGTGGGGGAGAACCATATATAAGTGCAGTAGTCTCTGTGAACATTCAAGCTTCTGCCTTCTCCCTCCTGTGAGTTTGGTAA
GTCACTGACTGTCTATGCCTGGGAAAGGGTGGGCAGGAGGTGGGGCAGTGCAGGAAAAGTGGCACTGTGAACCCTGCAGCCC
TAGACAATTGTACTAACCTTCTTCTCTTTCCTCTCCTGACAGGTTGGTGTACAGTAGCTTCCAAGGCCTAAGATCTCAGCTGCCAT
GGGCTCAATTGGTGCAGCATCAATGGAGTTCTGCTTTGATGTTTTCAAGGAGCTGAAAGTGCACCATGCAAATGAGAATATTTTT
TACTGCCCAATAGCAATAATGTCAGCCCTTGCTATGGTGTATCTGGGGGCCAAGGACTCCACCAGAACCCAAATCAACAAGGTT
GTAAGGTTTGACAAGCTGCCAGGCTTTGGTGACTCAATAGAGGCCCAGTGTGGCACCAGTGTAAATGTACACTCCTCCCTAAGG
GATATACTGAACCAGATAACCAAGCCCAATGATGTGTACAGCTTCTCCTTGGCAAGCAGACTATATGCAGAGGAGAGGTACCCA
ATCTTGCCTGAATACCTGCAGTGTGTCAAGGAACTTTACAGAGGGGGCCTAGAGCCCATCAACTTTCAGACTGCAGCTGACCAA
GCAAGGGAGTTAATCAACTCTTGGGTGGAGAGCCAGACCAATGGAATAATCAGGAATGTTCTGCAGCCTTCATCTGTAGACTCC
CAGACAGCAATGGTCTTGGTCAATGCAATTGTCTTCAAGGGCCTGTGGGAGAAGACTTTCAAAGATGAAGACACTCAGGCAATG
CCCTTCAGAGTAACTGAACAGGAGTCCAAACCTGTGCAGATGATGTACCAAATTGGGTTATTCAGGGTGGCTTCAATGGCTTCT
GAGAAAATGAAGATTCTGGAGTTACCCTTTGCCAGTGGGACAATGTCTATGCTGGTCCTGTTACCAGATGAGGTGTCAGGGCTT
GAGCAGCTGGAGTCAATCATCAATTTTGAGAAGTTAACAGAGTGGACCTCCTCCAATGTCATGGAAGAAAGGAAAATCAAGGTC
TACCTGCCCAGAATGAAAATGGAGGAGAAATACAACCTCACCTCAGTGTTGATGGCAATGGGGATAACAGATGTCTTCTCCAGC
TCTGCCAACCTCTCTGGCATCAGCAGTGCTGAATCCCTAAAGATATCACAGGCTGTTCATGCAGCCCATGCAGAAATCAATGAA
GCAGGCAGGGAGGTGGTGGGCTCTGCTGAGGCAGGAGTGGATGCTGCCTCTGTCTCAGAAGAGTTCAGAGCAGACCACCCCT
TCCTCTTCTGCATCAAGCATATAGCCACCAATGCTGTTCTTTTCTTTGGAAGGTGTGTGTCCCCCTAAAGCTAGCTGGCCAGACA
TGATAAGATACATTGATGAGTTTGGACAAACCACAACTAGAATGCAGTGAAAAAAATGCTTTATTTGTGAAATTTGTGATGCTATT
GCTTTATTTGTAACCATTATAAGCTGCAATAAACAAGTTAACAACAACAATTGCATTCATTTTATGTTTCAGGTTCAGGGGGAGGT
GTGGGAGGTTTTTTAAAGCAAGTAAAACCTCTACAAATGTGGTATGGAATTCAGTCAATATGTTCACCCCAAAAAAGCTGTTTGTT
AACTTGCCAACCTCATTCTAAAATGTATATAGAAGCCCAAAAGACAATAACAAAAATATTCTTGTAGAACAAAATGGGAAAGAATG
TTCCACTAAATATCAAGATTTAGAGCAAAGCATGAGATGTGTGGGGATAGACAGTGAGGCTGATAAAATAGAGTAGAGCTCAGA
AACAGACCCATTGATATATGTAAGTGACCTATGAAAAAAATATGGCATTTTACAATGGGAAAATGATGGTCTTTTTCTTTTTTAGAA
AAACAGGGAAATATATTTATATGTAAAAAATAAAAGGGAACCCATATGTCATACCATACACACAAAAAAATTCCAGTGAATTATAA
GTCTAAATGGAGAAGGCAAAACTTTAAATCTTTTAGAAAATAATATAGAAGCATGCCATCAAGACTTCAGTGTAGAGAAAAATTTC
TTATGACTCAAAGTCCTAACCACAAAGAAAAGATTGTTAATTAGATTGCATGAATATTAAGACTTATTTTTAAAATTAAAAAACCATT
AAGAAAAGTCAGGCCATAGAATGACAGAAAATATTTGCAACACCCCAGTAAAGAGAATTGTAATATGCAGATTATAAAAAGAAGT
CTTACAAATCAGTAAAAAATAAAACTAGACAAAAATTTGAACAGATGAAAGAGAAACTCTAAATAATCATTACACATGAGAAACTC
AATCTCAGAAATCAGAGAACTATCATTGCATATACACTAAATTAGAGAAATATTAAAAGGCTAAGTAACATCTGTGGCTTAATTAAA
ATCAGCAGTTCAACCTGTTGATAGTATGTACTAAGCTCTCATGTTTAATGTACTAAGCTCTCATGTTTAATGAACTAAACCCTCATG
GCTAATGTACTAAGCTCTCATGGCTAATGTACTAAGCTCTCATGTTTCATGTACTAAGCTCTCATGTTTGAACAATAAAATTAATAT
AAATCAGCAACTTAAATAGCCTCTAAGGTTTTAAGTTTTATAAGAAAAAAAAGAATATATAAGGCTTTTAAAGGTTTTAAGGTTTCC
TAGCTTTAGTCCTGTTCCTCAGCTACAAAATGGACACAATTTCCAGCAGGGTCTCTGAGGGCAAATTCCCTTCCCCAAGGTTGTT
CACCAATTTCTGTCATGGCTGGGCCAGAGGCATCCCTGAAATTTGTGCTGACTACTTCTGACCATTCTGCATAAAGCTCATCTAG
GCCTCTGACCCAGACCCAAGCAAGGGTGTTGTCAGGGACAACTTGGTCCTGAACTGCTGAGATGAAGAGGGTGACATCATCTC
TGACAACACCAGCAAAATCATCTTCAACAAAGTCTCTGGAGAATCCTAATCTGTCAGTCCAGAACTCTACAGCCCCTGCAACATC
CCTTGCTGTGAGGACTGGGACTGCAGAAGTGAGTTTGGCCATGATGGCCCTCCTATAGTGAGTTGTATTATACTATGCAGATATA
CTATGCCAATGTTTAATTGTCAACTACTGTT

Figure S1. Sequence data of pCpGfree-OVA(0)

The sequences of the open reading frame (OVA free from the unmethylated CpG motifs) are coloured in red.

Figure S2

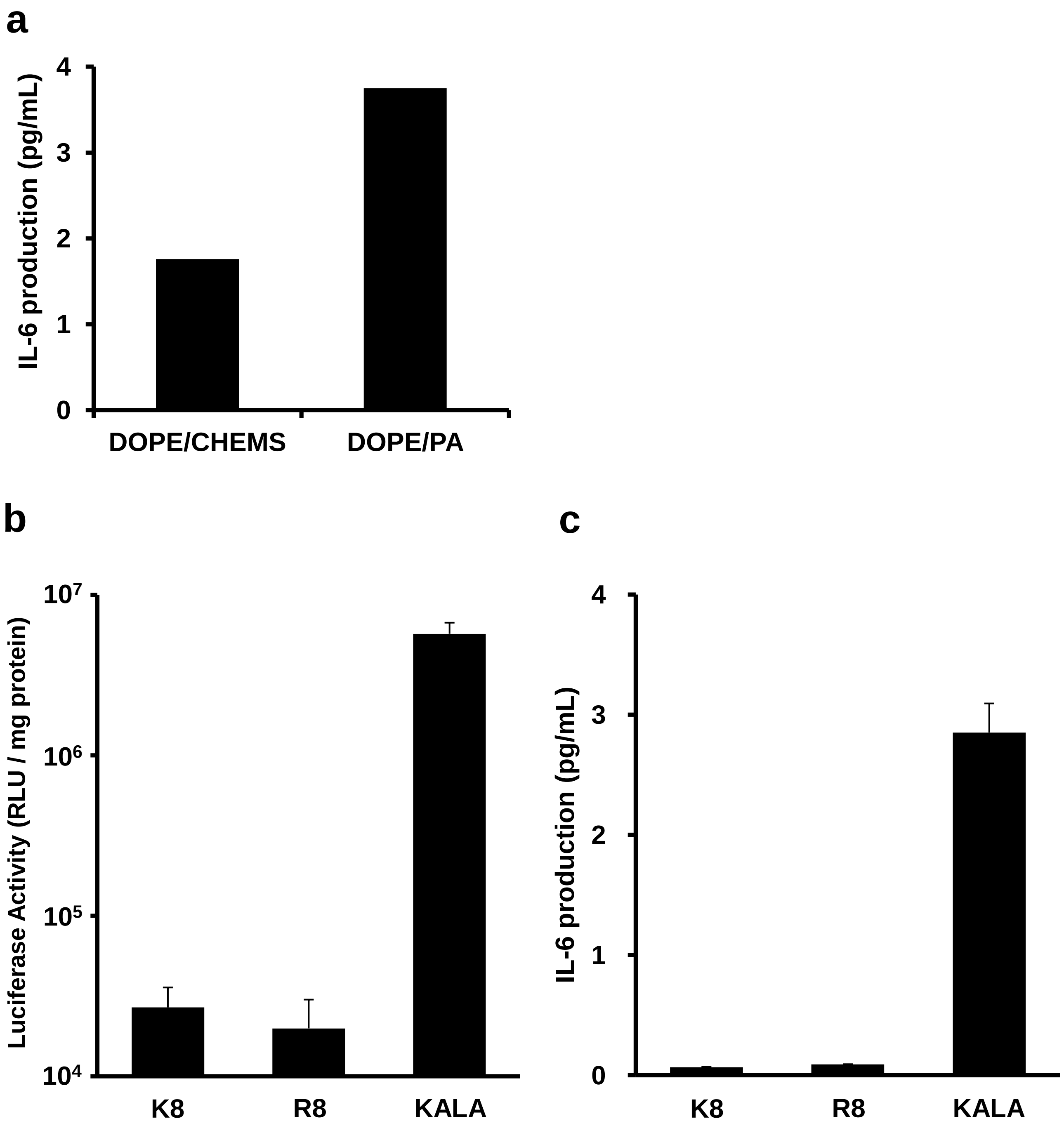

Figure S2. Effect of anionic lipid species and surface modification on gene transfection activity and IL-6 production

A. BMDCs (4.0×10^5 cells) were transfected with a KALA-MEND (equivalent to 0.4 μg pDNA) composed of DOPE/CHEMS or DOPE/PA. After 6 hours, the supernatant was recovered and the concentrations of IL-6 were measured by means of ELISA. **B.C.** The MENDs that were prepared with DOPE/PA was modified with stearylated KALA, R8 and K8. The MENDs were transfected to the BMDCs (4.0×10^5 cells) to evaluate the transfection activities after 24 h (**B**) or IL-6 production after 6 h (**C**).

Figure S3

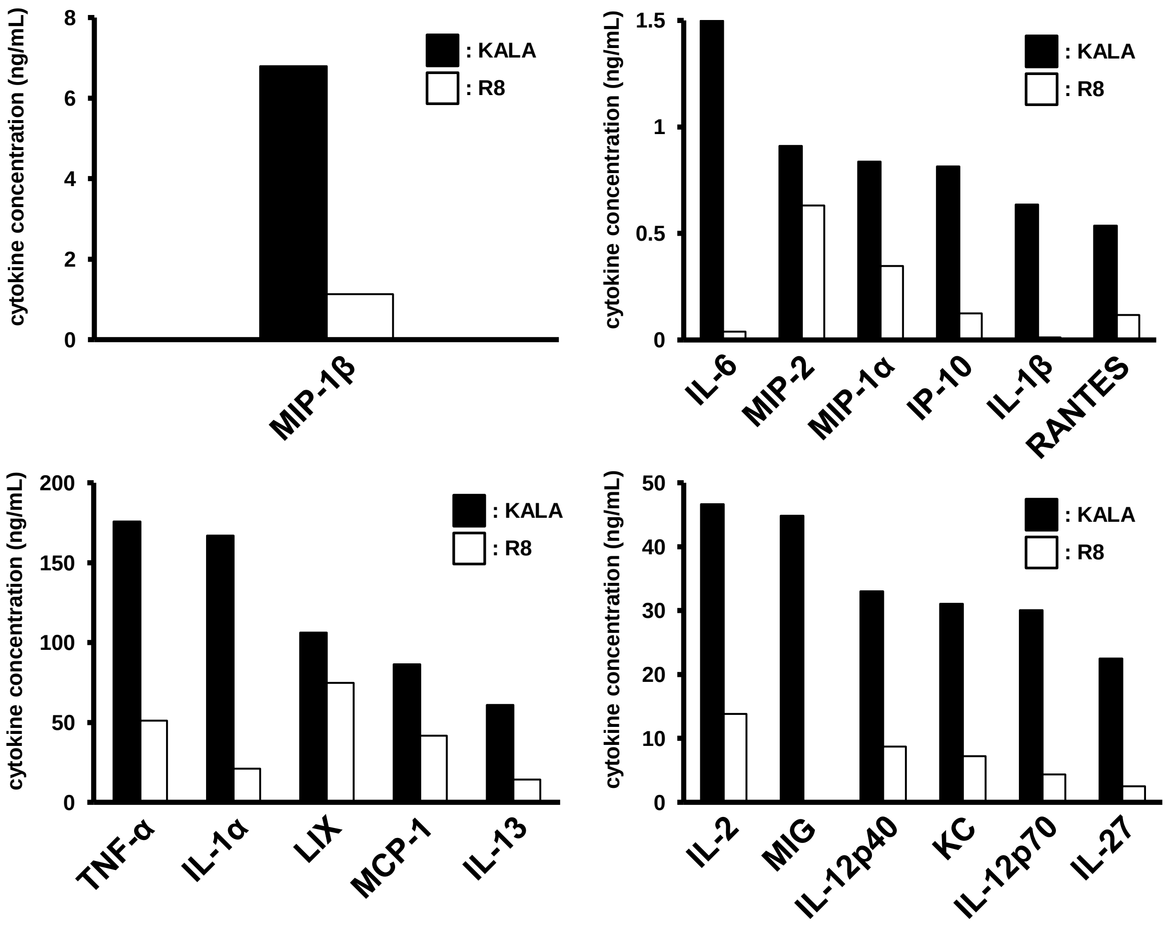

Figure S3. Comprehensive analysis of induced cytokines and chemokines

BMDCs (4.0×10^5 cells) were transfected with the KALA- or the R8-MEND (equivalent to 0.4 μg pDNA). After 6 hours, the supernatant was recovered and the concentrations of several cytokines, chemokines and other factors were measured by means of Multiplex Suspension Array System.

Figure S4

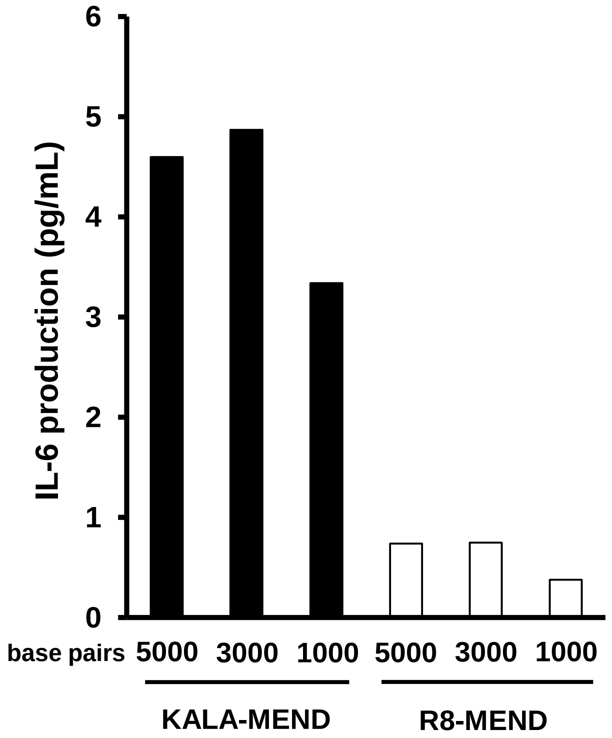

Figure S4. Effect of the length of the encapsulated DNA for IL-6 production

BMDCs (4.0×10^5 cells) were transfected with the KALA- or the R8-MEND (equivalent to 0.4 µg pDNA) encapsulating several types of DNA (approximately 1000, 3000 and 5000 base pairs).After 6 hours, the supernatant was recovered and the concentrations of IL-6 were measured by means of ELISA.

Figure S5

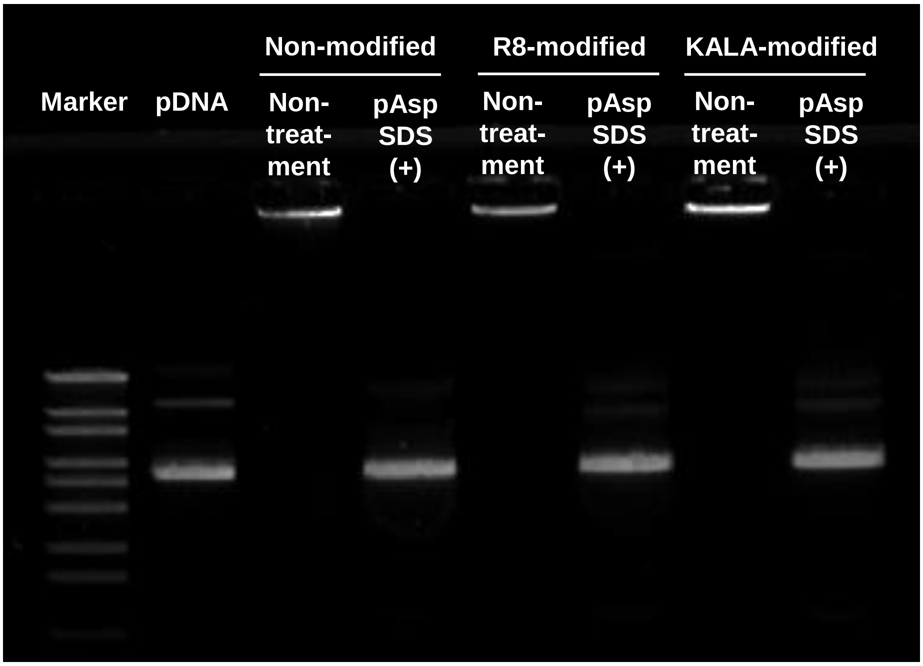

Figure S5. Association of pDNA with particle after the preparation.
The association of the pDNA with MENDs (non-modified, R8-modified and KALA-modified MEND) were evaluated by analysing the migration of pDNA through 1% agarose gel. The MENDs with or without SDS (1 % w/v) and poly-aspartic acid (pAsp, 1 mg/mL) was subject to gel electrophoresis, followed by staining with Gel Red™ (Biotium Inc., Hayward, CA, USA)

Figure S6

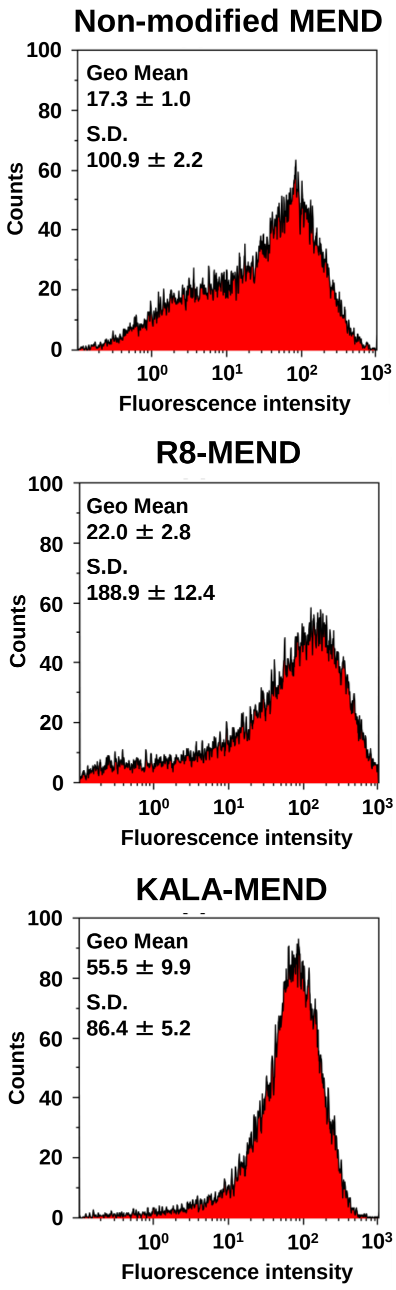

Figure S6. Cellular uptake of MENDs to BMDCs

For the analysis of the cellular uptake of non-modified MEND, R8-MEND and KALA-MEND, the particles were fluorescently labelled by adding the 0.02 mol% of DiO in the lipid composition. The BMDCs (1×10^6 cells) were incubated with the DiD-labeled MENDs (equivalent to 0.2 μg pDNA) for 1 hour. The cells were washed twice with ice-cold PBS. The cell suspension in the FACS buffer was filtered through a nylon mesh (45 μm mesh size) to remove cell aggregates, and then analyzed using a Gallios flow cytometer (Beckman Coulter, Inc., Brea, CA, USA).

Figure S7

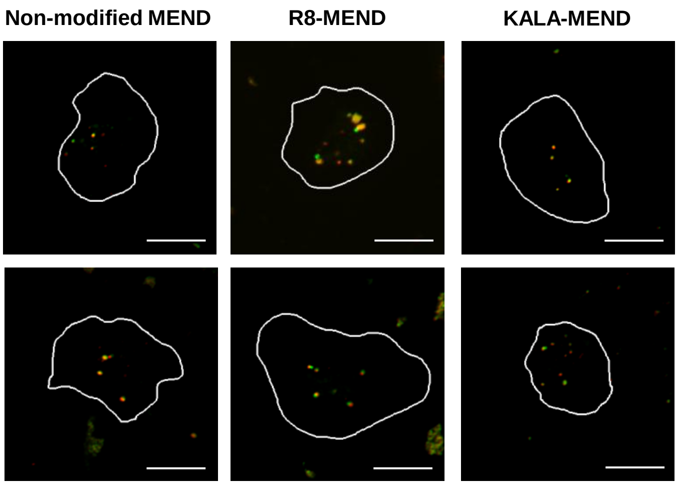

Figure S7. Visualization of the intracellular release of the pDNA from the MENDs by confocal microscopy

To evaluate the release of pDNA from the MENDs, BMDCs (4×10^5 cells) were incubated with DiO-labeld MENDs encapsulating QD705- pDNA (equivalent to 0.4 μg (KALA-MEND) or 1.2 μg (Non-modified MEND and R8-MEND) pDNA) on a glass base dish (Iwaki, Osaka, Japan) for 6 hrs in 1 ml of culture medium. After washing the cells 3 times with 1 ml portions of PBS, the culture medium was changed to HBSS and microscopic observations were performed. Confocal images were obtained using a Nikon ECLIPSE TE-2000-U equipped a spinning disk confocal unit, CSU-X1 and a Nikon Plan Apo 100×/1.40 oil immersion objective. The signals derived from QD705 and DiO are pseudocolored in red and green, respectively. Bars = 20 μm.
